# Biallelic Variants in *ASNA1*, Encoding a Cytosolic Targeting Factor of Tail-Anchored Proteins, Cause Rapidly Progressive Pediatric Cardiomyopathy

**DOI:** 10.1161/CIRCGEN.119.002507

**Published:** 2019-08-28

**Authors:** Judith M.A. Verhagen, Myrthe van den Born, Herma C. van der Linde, Peter G.J. Nikkels, Rob M. Verdijk, Maryann H. Kivlen, Leontine M.A. van Unen, Annette F. Baas, Henriette ter Heide, Lennie van Osch-Gevers, Marianne Hoogeveen-Westerveld, Johanna C. Herkert, Aida M. Bertoli-Avella, Marjon A. van Slegtenhorst, Marja W. Wessels, Frans W. Verheijen, David Hassel, Robert M.W. Hofstra, Ramanujan S. Hegde, Peter M. van Hasselt, Tjakko J. van Ham, Ingrid M.B.H. van de Laar

**Affiliations:** 1 Department of Clinical Genetics (J.M.A.V., M.v.d.B., H.C.v.d.L., L.M.A.v.U., M.H.-W., M.A.v.S., M.W.W., F.W.V., R.M.W.H., T.J.v.H., I.M.B.H.v.d.L.), Erasmus MC, University Medical Center Rotterdam.; 2 Department of Pathology (R.M.V.), Erasmus MC, University Medical Center Rotterdam.; 3 Department of Pediatric Cardiology (L.v.O.-G.), Erasmus MC, University Medical Center Rotterdam.; 4 Department of Pathology (P.G.J.N.), University Medical Center Utrecht, Utrecht University, the Netherlands.; 5 Department of Genetics (A.F.B.), University Medical Center Utrecht, Utrecht University, the Netherlands.; 6 Department of Pediatric Cardiology (H.t.H.), University Medical Center Utrecht, Utrecht University, the Netherlands.; 7 Department of Pediatrics (P.M.v.H.), University Medical Center Utrecht, Utrecht University, the Netherlands.; 8 Medical Research Council Laboratory of Molecular Biology, Cambridge Biomedical Campus, United Kingdom (M.H.K., R.S.H.).; 9 Department of Genetics, University of Groningen, University Medical Center Groningen, the Netherlands (J.C.H.).; 10 Centogene AG, Rostock (A.M.B.-A.).; 11 Department of Medicine III, University Hospital Heidelberg, Germany (D.H.).

**Keywords:** cardiomyopathies, endoplasmic reticulum, exome, membrane proteins, zebrafish

## Abstract

Pediatric cardiomyopathies are a clinically and genetically heterogeneous group of heart muscle disorders associated with high morbidity and mortality. Although knowledge of the genetic basis of pediatric cardiomyopathy has improved considerably, the underlying cause remains elusive in a substantial proportion of cases.

**Methods:**

Exome sequencing was used to screen for the causative genetic defect in a pair of siblings with rapidly progressive dilated cardiomyopathy and death in early infancy. Protein expression was assessed in patient samples, followed by an in vitro tail-anchored protein insertion assay and functional analyses in zebrafish.

**Results:**

We identified compound heterozygous variants in the highly conserved *ASNA1* gene (arsA arsenite transporter, ATP-binding, homolog), which encodes an ATPase required for post-translational membrane insertion of tail-anchored proteins. The c.913C>T variant on the paternal allele is predicted to result in a premature stop codon p.(Gln305*), and likely explains the decreased protein expression observed in myocardial tissue and skin fibroblasts. The c.488T>C variant on the maternal allele results in a valine to alanine substitution at residue 163 (p.Val163Ala). Functional studies showed that this variant leads to protein misfolding as well as less effective tail-anchored protein insertion. Loss of *asna1* in zebrafish resulted in reduced cardiac contractility and early lethality. In contrast to wild-type mRNA, injection of either mutant mRNA failed to rescue this phenotype.

**Conclusions:**

Biallelic variants in *ASNA1* cause severe pediatric cardiomyopathy and early death. Our findings point toward a critical role of the tail-anchored membrane protein insertion pathway in vertebrate cardiac function and disease.

## Abstract 2

Supplemental Digital Content is available in the text.

Dilated cardiomyopathy (DCM) is defined by otherwise unexplained ventricular dilatation and impaired systolic function, that can result in progressive heart failure, arrhythmias, and premature death.^1^ To date, disease-causing variants in over 30 genes have been reported in DCM; the majority encoding structural proteins of cardiomyocytes such as *TTN* (titin), *LMNA* (lamin A/C), and *MYH7* (myosin heavy chain 7).^2^ The same genes that are involved in adult-onset DCM also contribute to pediatric DCM, although the exact frequencies are unclear.^3,4^ De novo variants or a combination of multiple inherited variants may explain early-onset and severe disease presentation.^3,5^ Pediatric DCM can also be part of numerous syndromes and neuromuscular or metabolic disorders. However, the underlying cause remains unknown in ≈50% of cases.^6,7^

Here, we used family-based exome sequencing and subsequent functional validation to identify compound heterozygous variants in *ASNA1* in 2 siblings with early infantile-onset, rapidly progressive DCM. ASNA1, also known as TRC40 or GET3, is a ubiquitously expressed cytosolic chaperone that mediates insertion of TA (tail-anchored) proteins into the endoplasmic reticulum (ER) membrane.^8^ TA proteins are membrane proteins characterized by a single hydrophobic transmembrane domain near the C-terminus which serves as both a targeting signal and a membrane anchor.^9^ TA proteins constitute ≈5% of integral membrane proteins and are involved in a variety of cellular processes, such as protein translocation, vesicle trafficking, and apoptosis.^10^ Previous animal studies have implicated ASNA1-mediated membrane insertion of TA proteins in early embryonic development.^11–16^ This study offers the first evidence for its role in human disease, and provides new insight into the molecular mechanisms in DCM.

## Methods

The authors declare that all supporting data are available within the article and its in the Data Supplement. Affected individuals were recruited from 3 clinical genetic centers in the Netherlands. All samples were collected after obtaining informed consent in compliance with clinical research protocols approved by the local institutional review boards. Zebrafish (*Danio rerio*) were raised and maintained under standard conditions.^17^ All zebrafish experiments were performed in compliance with Dutch animal welfare legislation. Study protocols were approved by the institutional review board for experimental animals. Details on the materials and methods are available in the Data Supplement (including Tables I and II and Figures I and II in the Data Supplement).

## Results

### Clinical Presentation

**Figure 1.**
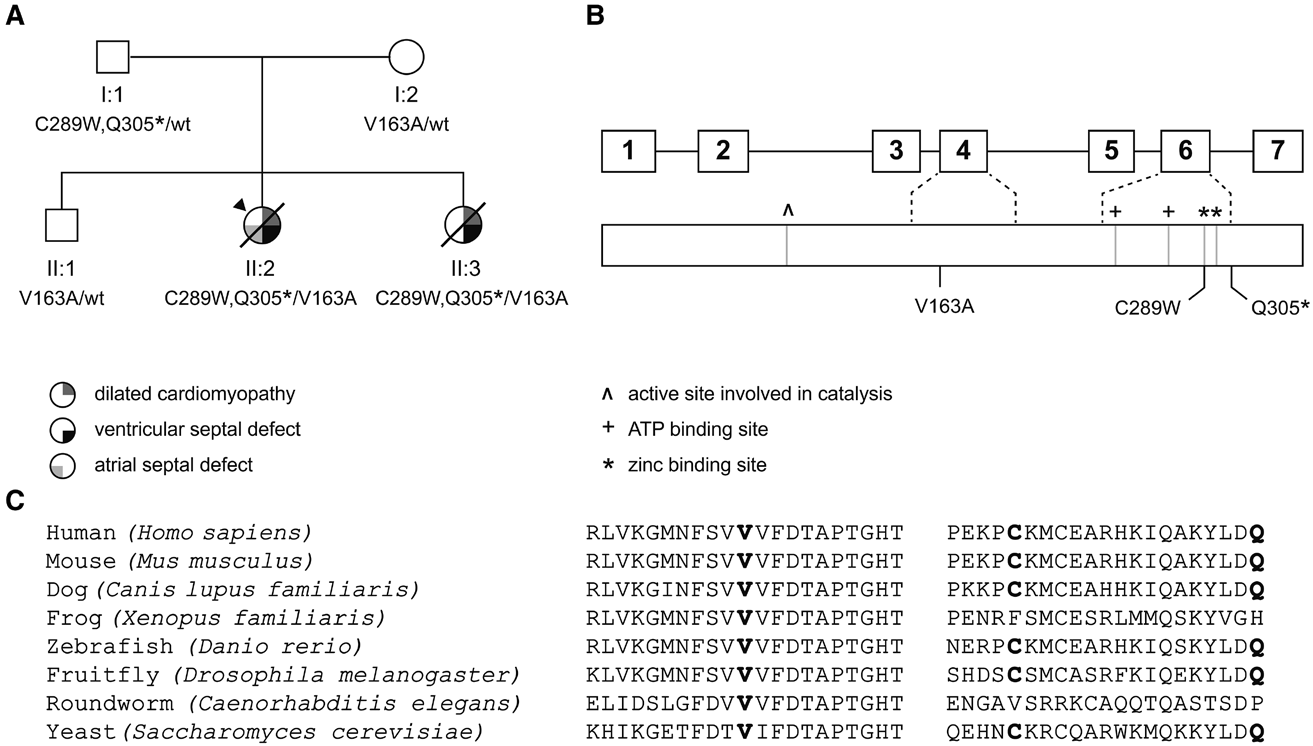
**Pedigree, ASNA1 and pathogenic variants.** A, Pedigree of the family. Squares and circles indicate males and females, respectively. The arrowhead indicates the proband. Upper right symbols indicate dilated cardiomyopathy, lower right symbols indicate ventricular septal defect, and lower left symbol indicates atrial septal defect. Genetic status is displayed below the symbols: wt, wild-type. **B**, Schematic overview of the *ASNA1* gene (**top**) and corresponding protein (**bottom**). Boxes represent exons; connecting lines represent intervening introns. ^ indicates active site involved in catalysis, + indicates ATP binding sites, and * indicates zinc-binding sites. Rare variants identified in our family are displayed at the bottom of the diagram. **C**, Ortholog alignment of ASNA1 (derived from Ensembl reference sequences), showing high degree of protein conservation across vertebrates. Positions of disease-causing variants discovered in our family are indicated in bold.

**Figure 2.**
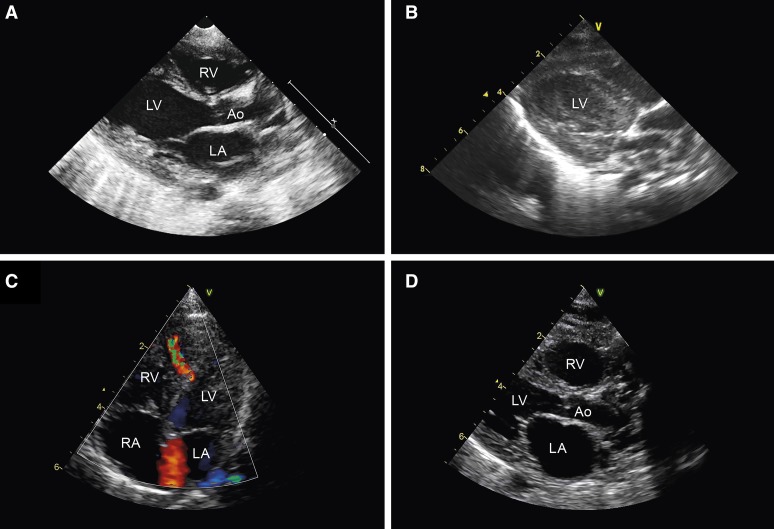
**Cardiac ultrasound examination.** Patient II:2 (**A**) parasternal long-axis view during extracorporeal membrane oxygenation (ECMO) showing mild dilatation of the left ventricle; (**B**) intracardiac thrombus formation. Note: images from the echocardiogram made before ECMO were not available. Patient II:3 (**C**) 4-chamber view at first day postpartum showing a midmuscular ventricular septal defect. **D**, Parasternal long-axis view during cardiopulmonary resuscitation showing dilatation of the heart chambers. Ao indicates aorta; LA, left atrium; LV, left ventricle; RA, right atrium; and RV, right ventricle.

The proband (Figure 1A: II:2) was the second child of nonconsanguineous white parents, born at term after an uneventful pregnancy. At age 2 weeks, she presented with severe tachypnea and feeding difficulties. No dysmorphic features were observed. Echocardiography revealed a small muscular ventricular septal defect, an ostium secundum atrial septal defect, and impaired left ventricular (LV) contractility (LV ejection fraction 41%; Figure IIIA in the Data Supplement). ECG recordings showed sinus rhythm with broad QRS complexes (range 124–264 ms; Figure IVA in the Data Supplement). After rapid clinical deterioration with brief circulatory arrest, she was transferred to a tertiary referral hospital for extracorporeal membrane oxygenation. LV function remained poor without any signs of improvement (Figure 2A). In addition, a large LV thrombus developed refractory to medical therapy (Figure 2B, Movie I in the Data Supplement). The patient died at age 7 weeks.

Her younger sister (II:3) was born at term after an uneventful pregnancy with normal second-trimester advanced ultrasound examination. Because of the family history, echocardiography was performed at the first day postpartum showing a small midmuscular ventricular septal defect but otherwise normal size and function of the heart (Figure 2C, Figure IIIB in the Data Supplement). She was reexamined after a week because of tachypnea. Echocardiographic findings were essentially unchanged (Movie II in the Data Supplement). However, only 3 days later (age 12 days), she presented with cardiorespiratory failure necessitating resuscitation. Echocardiography now showed dilatation of the heart chambers with poor contractility (Figure 2D and Movie III in the Data Supplement). ECG recordings in the resuscitation setting were severely abnormal (Figure IVB in the Data Supplement). The resuscitation attempt was terminated after 20 minutes.

In both siblings, extensive biochemical, hematologic, viral, and metabolic testing was unremarkable except for slightly abnormal serum transferrin and apolipoprotein C-III isoelectric focusing profiles, indicative of a combined defect in N-linked and O-linked glycosylation. Cardiac screening in both parents (aged 36 and 37 years) and the elder brother (aged 34 months), consisting of physical examination, 12-lead ECG and echocardiography, revealed no abnormalities. A full 3-generation family history was negative for cardiomyopathy, heart failure and sudden cardiac death.

### Exome Sequencing

Targeted next-generation sequencing of 48 genes implicated in cardiomyopathy revealed no potentially deleterious variants.^18^ Exome sequencing in the affected proband (II:2) and her healthy parents identified 3 novel heterozygous variants in *ASNA1* (NM_004317.2): 2 variants c.867C>G p.(Cys289Trp) and c.913C>T p.(Gln305*) in *cis* configuration on the paternal allele, and a missense variant c.488T>C p.(Val163Ala) on the maternal allele (Figure 1A and 1B). No other potentially deleterious variants were detected. We confirmed that the affected sister (II:3) carried all 3 *ASNA1* variants. The unaffected brother (II:1) had inherited only the maternally derived *ASNA1* variant (Figure 1A). All variants were absent from public databases, including the nearly 140 000 alleles in gnomAD v2.0.2. The high pLI score (0.92) indicates that *ASNA1* is extremely intolerant to loss-of-function variants. Both missense variants were predicted to be deleterious (CADD >20 and M-CAP >0.025). The c.913C>T variant introduces a premature stop codon, likely resulting in the loss of the last 42 amino acids. In silico, analysis did not predict an effect on splicing using the nearby splice site. Reverse transcription-polymerase chain reaction analysis showed equal amounts of wild-type and mutant products. Hence, no evidence was found for nonsense-mediated decay. No alternatively spliced transcripts were detected (data not shown).

### Cohort Screening

To find additional cases, we sequenced 70 children with idiopathic cardiomyopathy for *ASNA1* variants using either Sanger sequencing or filtering of exome sequencing data. No biallelic variants were found. In one patient, presenting with severe DCM requiring heart transplantation at age 16 years, we identified one paternally inherited, heterozygous missense variant c.547G>A p.(Val183Met) in *ASNA1*. Genome-wide microarray analysis excluded a large deletion of the second allele. However, in addition, a de novo disease-causing variant c.473T>C p.(Leu158Pro) was found in *LMNA* (NM_17070.2), generally associated with adult-onset DCM. Although the *ASNA1* variant is rare and assigned to the top 1% most deleterious substitutions possible in the human genome (CADD score 23.1), it is predicted to be tolerated by Sorting Intolerant From Tolerant and PolyPhen-2, and classified as likely benign by M-CAP. Nevertheless, given the relatively early-onset and severe disease presentation, it cannot be excluded that this *ASNA1* variant acted as a modifier of the *LMNA*-related cardiomyopathy. A second search aiming to identify further patients was performed in Centogene’s internal database, which contains next-generation sequencing data from a heterogeneous cohort of 19 144 index patients with suspected genetic diseases and a total of 33 762 samples (as per July 2018). However, no additional patients were identified with rare biallelic variants in *ASNA1*.

### Histopathologic Examination

**Figure 3.**
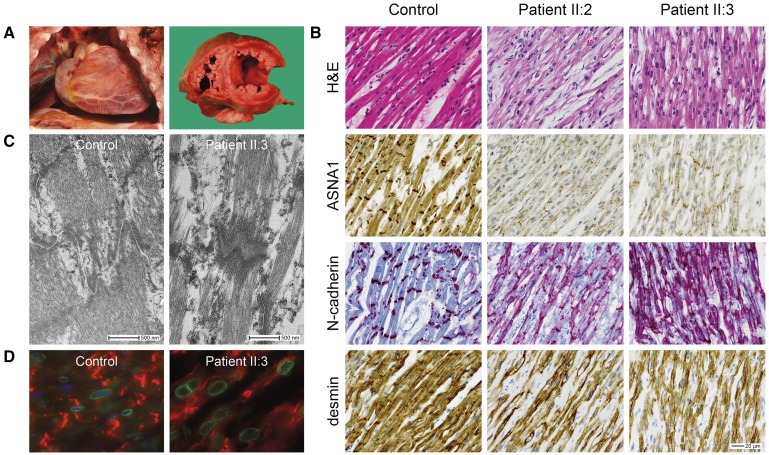
**Histopathologic features of the myocardium.** **A**, Macroscopic images showing an enlarged heart with dilated left ventricle in patient II:3. **B**, Representative images of histological and immunohistochemical studies of myocardial tissue showing markedly reduced expression of ASNA1 at the cytoplasm and intercalated discs in both patients compared with an age-matched control. N-cadherin staining showing irregular appearance of the intercalated discs. Desmin staining showing myofibrillar disorganization. Scale bar: 20 µm. **C**, Electron microscope images of cardiac intercalated discs, showing increased intercellular space in the patient compared with an age-matched control. **D**, Representative images of immunofluorescence double-staining of emerin (nuclear membrane, green fluorescence) and N-cadherin (intercalated disc, red fluorescence) in myocardial tissue of patient and control. Note irregular nuclear shape in the patient. Note: experiments were performed in both patients. However, as the images of patient II:2 were of too low quality for publication, only images from patient II:3 are displayed here.

In both siblings, postmortem examination revealed an increased heart weight to body size and severe dilatation of the LV (Figure 3A). No other gross abnormalities were observed. Microscopic examination of the myocardium showed prominent subendothelial fibrosis. In age-matched controls, ASNA1 was predominantly localized to the cytoplasm and intercalated discs. Though subcellular localization of ASNA1 appeared unchanged, expression was markedly reduced in both patients compared with controls (Figure 3B). As demonstrated by N-cadherin labeling (Figure 3B) and electron microscopy (Figure 3C), in both patients, intercalated discs were irregular in appearance and intercellular space was increased. Desmin staining confirmed myofibrillar disorganization (Figure 3B). We examined the subcellular localization of the TA protein emerin in myocardium using immunofluorescence staining. Emerin correctly localized to the nuclear membrane. However, nuclei had an irregular shape (Figure 3D). Microscopic examination of other visceral organs did not reveal any obvious abnormalities (data not shown).

### Biochemical Analysis of ASNA1 Protein

**Figure 4.**
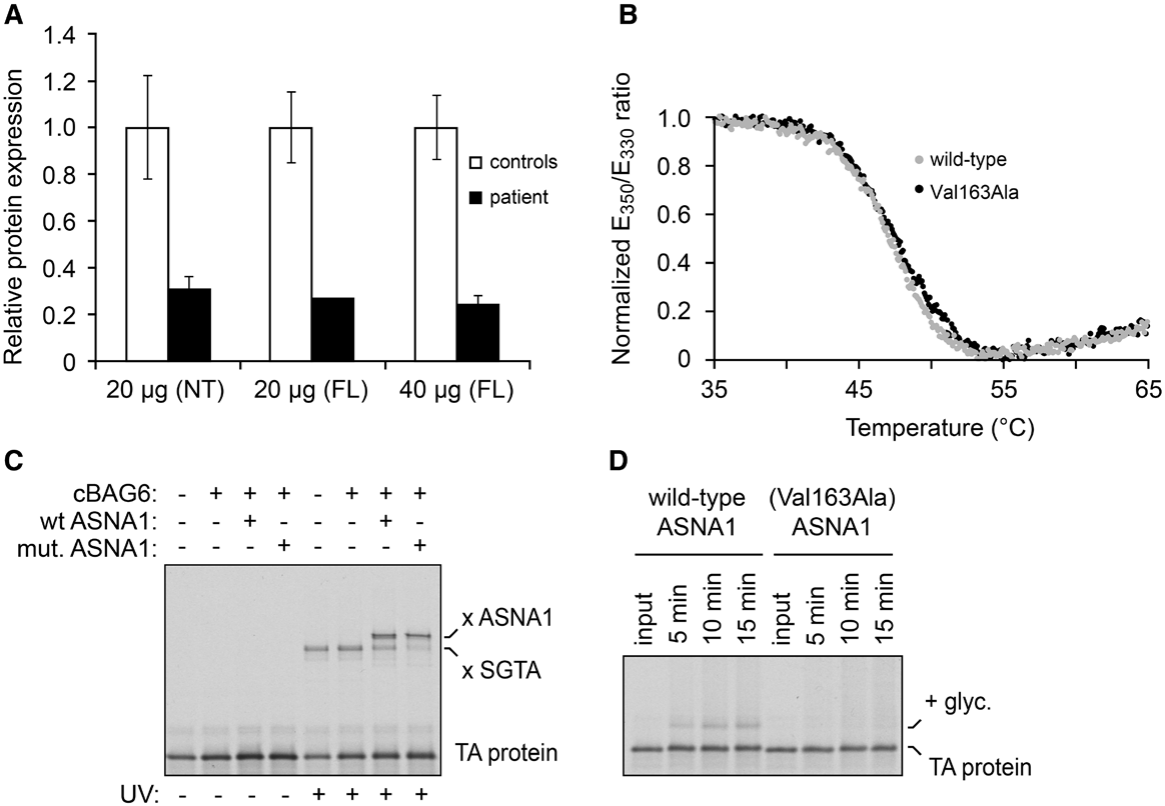
**Biochemical analyses of ASNA1.** **A**, Expression levels of ASNA1 protein in skin fibroblasts from patient II:2 compared with healthy controls, and normalized against GAPDH as measured by Western blot analysis. Relative expression is expressed as mean±SD from 1 to 3 different experiments. Error bars represent SD. **B**, Thermal unfolding curves of purified recombinant wild-type and Val163Ala mutant ASNA1. The ratio of tryptophan fluorescence emission at 350 nm to 330 nm was measured during a temperature ramp. The ratio was normalized using the highest and lowest values and scaled to 1.0 and 0, respectively. This ratio is sensitive to the environment around the tryptophan, and therefore changes during protein unfolding. Both wild-type and mutant ASNA1 unfold at the same temperature (between 45°C and 50°C), indicating that they are comparably stable. **C**, Radiolabeled TA (tail-anchored) protein assembled on the chaperone Small Glutamine Rich Tetratricopeptide Repeat Containing Alpha (SGTA) was mixed with wild-type or mutant ASNA1 together with the cBAG6 (complement BCL2 Associated Athanogene 6) complex (which bridges SGTA and ASNA1), incubated for 90 seconds, and subjected to UV-induced crosslinking. In the reaction lacking ASNA1, the TA protein crosslinks to SGTA (x SGTA) in a UV-dependent manner. Transfer from SGTA to ASNA1 (as evidenced by crosslinking to ASNA1 after incubation) is observed for wild-type and Val163Ala mutant ASNA1. **D**, Radiolabeled TA protein in complex with either wild-type or Val163Ala mutant ASNA1 (Figure VD in the Data Supplement) was incubated with ER microsomes for the indicated times. Input indicates an aliquot of the starting complex analyzed for comparison. ER insertion was monitored by the appearance of a glycosylated form of the TA protein (indicated by + glyc). Insertion is less efficient for the reactions containing mutant ASNA1. FL indicates full-length; and NT, N-terminus.

As expected from the reduced ASNA1 expression by immunohistochemistry (Figure 3B), Western blot experiments confirmed that ASNA1 was decreased in fibroblasts of patient II:2 (Figure 4A), suggesting that mutant ASNA1 protein in this patient is unstable. This is to be expected for the (Cys289Trp;Gln305*) double mutant. The Cys289 variant is part of an essential zinc-binding site; residues downstream of Gln305 would be essential for structural integrity of ASNA1.^19^ The other mutated residue, Val163, is universally conserved from yeast Get3 to human ASNA1 and forms part of the hydrophobic domain,^19^ suggesting that substitution of this residue might also lead to reduced stability and function of the protein. To explore this possibility, we investigated the consequences of the Val163Ala variant in vitro using recombinant zebrafish ASNA1 protein.

Although Val163Ala mutant ASNA1 was expressed equally well as wild-type ASNA1 in *E. coli*, the mutant was mostly insoluble indicating its inefficient folding (Figure VA in the Data Supplement). The folded population of mutant ASNA1 was purified (Figure VB in the Data Supplement) and shown to display comparable thermal stability as wild-type ASNA1 (Figure 4B). Recombinant mutant ASNA1 was also comparably efficient as wild-type ASNA1 in capturing a TA protein substrate (Figure 4C) using a previously established in vitro assay.^20^ However, TA protein in complex with mutant ASNA1 was very poorly inserted into ER microsomes compared with TA protein in complex with wild-type ASNA1 (Figure 4D). Thus, the Val163Ala variant has 2 consequences. First, it reduces the production of folded ASNA1 due to aggregation. Second, properly folded mutant ASNA1, while competent for TA protein interaction, is inefficient in facilitating TA protein insertion into the ER membrane.

### Zebrafish Mutants

**Figure 5.**
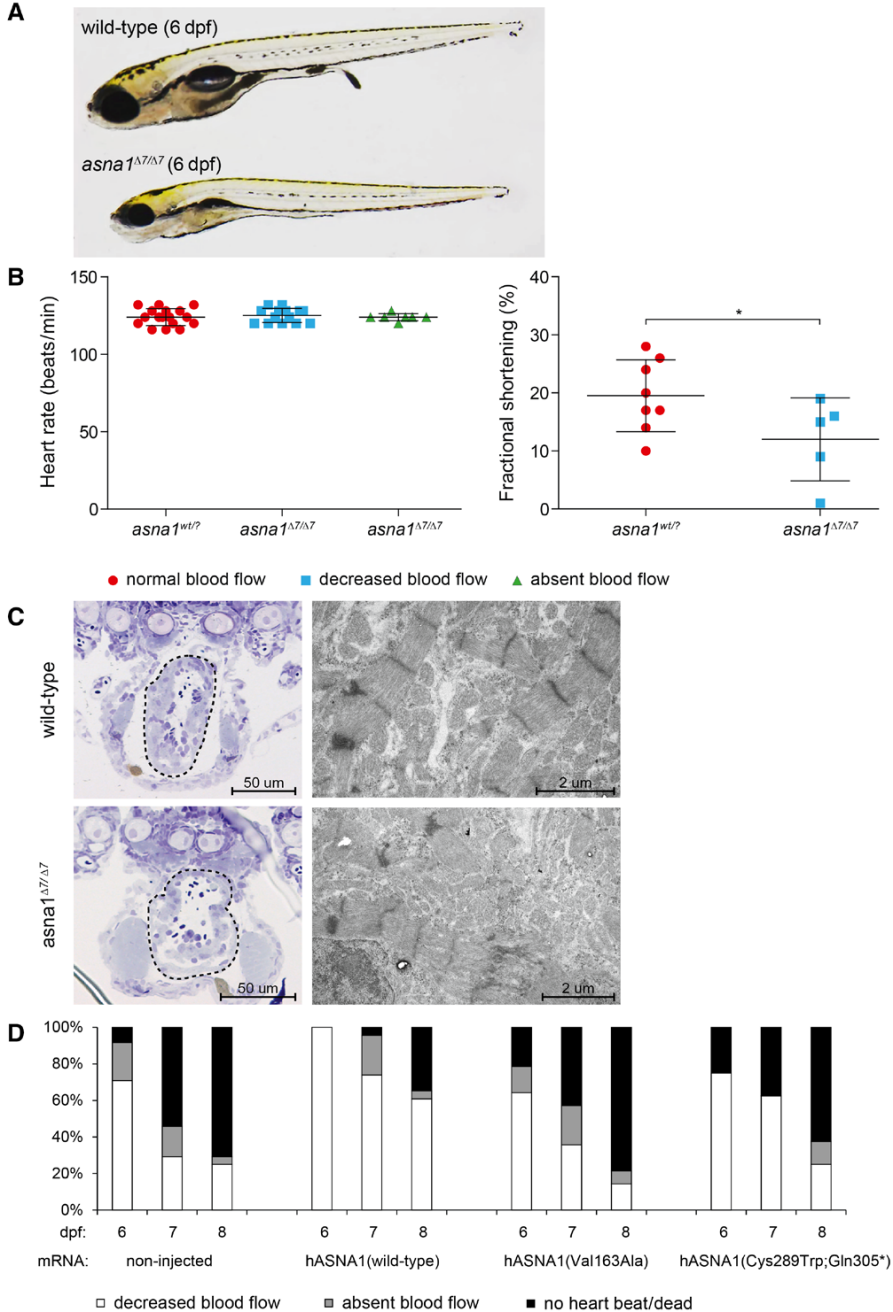
**Knockout of asna1 causes cardiac failure in zebrafish larvae.** **A**, Lateral view of wild-type and homozygous *asna1* mutant (*asna1*^Δ7/Δ7^) larvae at 6 dpf. Overall, *asna1*^Δ7/Δ7^ mutants did not show an overt embryonic phenotype besides smaller body size and lack of swim bladder inflation. **B**, Quantification of cardiac function in zebrafish larvae. Mean heart rate (beats per minute) did not differ between the groups, even in the absence of blood flow. Fractional shortening was significantly reduced in *asna1*^Δ7/Δ7^ mutants compared with wild-type and heterozygous clutchmates (*P*=0.0349). **C**, Microscopic imaging of zebrafish hearts. Coronary sections (**left**) showing abnormal shaped ventricle with thinner wall in *asna1*^Δ7/Δ7^ mutant. Electron microscopy images (**right**) showing myofibrillar disorganization and abnormal intercalated disc ultrastructure in *asna1*^Δ7/Δ7^ mutant. **D**, From 5 dpf, *asna1*^Δ7/Δ7^ mutants showed reduced to absent red blood cell flow rate. At 9 dpf, all *asna1*^Δ7/Δ7^ mutants had died. Injection of wild-type human *ASNA1* mRNA significantly ameliorated the phenotype (*P*<0.005 at all time points), whereas injection of either mutant (Val163Ala or Cys289Trp;Gln305*) mRNA had no significant effect.

The zebrafish gene and protein share 82% and 95% sequence identity with their respective human counterparts. To confirm the role of *ASNA1* variants in cardiac disease, we generated *asna1*-deficient mutant zebrafish by Clustered Regularly Interspaced Short Palindromic Repeats/Cas9-mediated genome editing. Incrossed heterozygous *asna1* mutants (*asna1*^Δ7/*+*^) resulted in Mendelian ratios of progeny. On gross examination, *asna1*^Δ7/Δ7^ embryos had impaired swim bladder inflation and smaller body size compared with their wild-type and heterozygous clutchmates (Figure 5A). From 5 dpf, *asna1*^Δ7/Δ7^ mutants displayed abnormal cardiac contractions and decreased blood flow velocity in the dorsal aorta and cardinal vein (Movies IV and V in the Data Supplement). Fractional shortening was significantly reduced in *asna1*^Δ7/Δ7^ mutants compared with wild-type and heterozygous clutchmates (*P*=0.0349). Mean heart rate was not significantly different between all groups, even after cessation of blood flow (Figure 5B), pointing towards a primary defect in cardiac contractility and not the electrical system. Compatible with the findings in our family, heterozygous mutants (*asna1*^Δ7/*+*^) did not show any overt phenotype. In contrast, none of the homozygous mutants (*asna1*^Δ7/Δ7^) survived past 8 dpf.

On microscopic examination, hearts of *asna1*^Δ7/Δ7^ zebrafish were irregular in shape and had thinner walls compared with wild-type and heterozygous clutchmates. In addition, electron microscopic examination revealed less organized Z-lines (plate-like structures that anchor actin filaments) and irregular intercalated discs in *asna1*^Δ7/Δ7^ zebrafish (Figure 5C). Injection of wild-type human *ASNA1* mRNA into *asna1*^Δ7/Δ7^ zebrafish embryos significantly rescued the phenotype at each time point examined (*P*<0.0005; Figure 5D). For example, at 8 dpf only 38% of fish that were injected with wild-type human *ASNA1* mRNA had died or showed absent blood flow compared with 75% of noninjected fish. This rescue effect seems to disappear over time, likely due to mRNA degradation. In contrast, to rescue observed with wild-type *ASNA1* mRNA, injection of either the paternal or maternal mutant *ASNA1* mRNA failed to rescue the disease phenotype (Figure 5D), supporting their pathogenicity.

### Key Candidate Proteins

Inspection of the list of predicted human TA proteins (Table III in the Data Supplement) revealed 7 proteins of interest that have been associated with cardiomyopathy in humans: DMPK (myotonin-protein kinase; Q09013), DYSF (dysferlin; O75923), EMD (emerin; P50402), JPH2 (junctophilin-2; Q9BR39), PPLA (cardiac phospholamban; P26678), SYNE1 (nesprin-1; Q8NF91), and SYNE2 (nesprin-2; Q8WXH0).

## Discussion

Our results show that biallelic loss-of-function variants in *ASNA1* cause a rapidly progressive cardiomyopathy resulting in acute heart failure and death in early infancy. We report that *asna1* deficiency in zebrafish also causes cardiac defects and early lethality, which implies that, in vertebrates, the TA protein insertion pathway is specifically critical to development and function of the heart. ASNA1 binds to the transmembrane segment of newly synthesized TA proteins and delivers them to the WRB/CAML receptor complex for insertion into the ER membrane.^21^ Together, this complex is essential for efficient and proper targeting of a wide range of TA proteins.^22^ Thus far, the corresponding genes *ASNA1* (MIM 601913), *WRB* (MIM 602915), and *CAMLG* (MIM 601118) have not been associated with disease in humans.

The nucleotide and amino acid sequences of ASNA1 are highly conserved across vertebrate species (Figure 1C). The mouse *Asna1* gene and corresponding protein share 90% nucleotide identity and 100% amino acid identity with its human counterparts. Homozygous *Asna1* knockout mice, though apparently normal at the blastocyst (E3.5) stage, displayed early embryonic lethality.^11^ Heterozygous *Asna1* knockout mice, on the contrary, were viable and showed no apparent abnormalities. These findings underscore that *Asna1* plays a crucial role in embryonic development, and that one functional copy of the gene is sufficient for normal development. The prevalence of *ASNA1*-related cardiomyopathy is probably low, given the negative results upon cohort screening (n=70) and the low rate of protein-altering variants in population data sets. Considering the rapidly fatal disease course, additional patients may be found in cases of sudden unexpected infant death, or, assuming that severe impairment of ASNA1 is incompatible with life,^11^ families with recurrent miscarriage or fetal death.

We explored the role of *asna1* in cardiac development in the zebrafish. Unlike mice, zebrafish embryos are not dependent on a functional cardiovascular system for sufficient supply of oxygen but rely on passive diffusion.^23^ Embryos with severe cardiovascular defects can, therefore, be studied past the initial stages of embryonic development. Here, we used Clustered Regularly Interspaced Short Palindromic Repeats/Cas9-mediated genome editing to generate a loss-of-function model for *ASNA1* in zebrafish. This strategy resulted in an early cardiac phenotype. Clustered Regularly Interspaced Short Palindromic Repeats-mediated *asna1*^Δ7/Δ7^ knockouts displayed decreased blood flow in the dorsal aorta, impaired cardiac contractility, and premature lethality, recapitulating the heart failure phenotype observed in our patients.

Previous studies in vertebrate models of the WRB-CAMLG receptor complex also point toward a role in cardiac development and disease (Table IV in the Data Supplement). Morpholino knockdown of *wrb* in clawed frogs (*Xenopus tropicalis*) and medaka fish (*Oryzias latipalis*) induced cardiac looping defects and abnormal chamber differentiation.^12,14^ Of note, microscopic analysis in *wrb*-deficient frogs revealed large intercellular gaps between cardiomyocytes, reminiscent of the intercalated disc abnormalities observed in our family. These findings suggests that genes encoding other components of the TA protein insertion pathway may be good candidate genes for cardiovascular disease as well.

The exact mechanism by which *ASNA1* variants result in cardiomyopathy remains to be determined. Several TA proteins have been linked to cardiomyopathy (including dysferlin, emerin, junctophilin-2, phospholamban, nesprin-1, and nesprin-2), and failure to correctly localize one or more of these proteins, due to defective ASNA1-mediated membrane insertion, may be responsible for the cardiac phenotype observed in both patients and zebrafish. Intriguingly, variants in the gene *EMD*, which cause a progressive skeletal muscle weakness and cardiomyopathy known as X-linked Emery-Dreifuss muscular dystrophy (MIM 310300), result in mislocalization of the protein due to impaired ASNA1-mediated nuclear targeting.^24^ Though emerin staining showed apparently normal localization of the protein in our patients, it did reveal the characteristic abnormal nuclear morphology previously described in Emery-Dreifuss muscular dystrophy.^25^ Similarly, variants in the *PLN* (phospholamban) gene, which can result in various types of cardiomyopathy (MIM 609909 and 613874), impair proper localization of cardiac phospholamban to the ER membrane.^26^ Interestingly, patients with *PLN*-associated heart disease also exhibit intercalated disc abnormalities.^27^ Taken together, we hypothesize that defective ASNA1-mediated targeting affects several cardiomyopathy-related TA proteins, which together may explain the early-onset and severity of disease in our patients.

Given the ubiquitous expression of ASNA1 and the fundamental cellular processes TA proteins are involved in, one might expect that biallelic loss-of-function variants in *ASNA1* would have more pleiotropic effects. Indeed, zebrafish mutant for Asna1 or for the Asna1 receptor Wrb have visual function defects and reduced touch response (Table IV in the Data Supplement).^28,29^ In addition, both mouse and zebrafish Wrb mutants have hearing defects due to mislocalization of the TA protein otoferlin, indicating the ASNA1-mediated TA protein insertion is critical in hearing.^29,30^ Moreover, in the nematode *Caenorhabditis elegans*, reduced *asna1* activity causes exocytosis defects leading to defective insulin secretion, which was confirmed in pancreatic mouse *Asna1* knockouts.^15,16^ While no extra-cardiac abnormalities were found in the siblings it is possible that other abnormalities have gone unnoticed, did not yet develop at this early age, or were masked by the low but detectable functionality of the Val163Ala mutant protein. Of note, both siblings had passed the newborn hearing screening.

A distinct subset of TA proteins are involved in vesicular trafficking between the ER and Golgi and the secretory machinery, including several SNAREs (SNAP-receptors) and VAMPs (vesicle-associated membrane proteins) essential for intracellular membrane fusion (Table III in the Data Supplement). Glycosylation of proteins and lipids, a complex process which starts in the ER and continues in the Golgi, highly depends on intracellular vesicular trafficking. Therefore, it is possible that the abnormal isoelectric focusing profiles of transferrin and apolipoprotein C-III in both our patients result from defective membrane targeting of TA proteins involved in vesicular trafficking and exocytosis. On the contrary, as isoelectric focusing profiles were only slightly abnormal, these results should not necessarily be considered pathogenic.

TA proteins do not solely rely on ASNA1 for insertion into the ER membrane. A subset of TA proteins with moderately hydrophobic transmembrane domains can integrate in the ER membrane via an alternative route dependent on the highly conserved EMC (ER membrane protein complex).^31^ Although other routes have been demonstrated in vitro,^32,33^ their functional contribution to TA protein insertion in mammalian cells remains unclear at present. These alternative routes might not be effective or sufficient for all TA proteins, in particular strongly hydrophobic TA proteins (such as the vesicle-associated membrane protein 2), or in all cell types, suggesting why certain proteins or tissues might be more severely affected by defective ASNA1-mediated targeting.

Taken together, our study shows that biallelic variants in *ASNA1*, encoding a cytosolic targeting factor for TA proteins, cause severe pediatric DCM with early-onset and rapid progression. We hypothesize that this phenotype is caused by mislocalized TA proteins, either by toxic aggregation or reduced levels of functional protein. Our findings point toward a critical role of the TA protein insertion pathway in vertebrate heart function and disease.

## Acknowledgments

We wish to thank the family members for their participation in this study. We thank Tom de Vries Lentsch (Department of Clinical Genetics, Erasmus MC, University Medical Center Rotterdam) for the photographic artwork, Mike Broeders (Department of Clinical Genetics, Erasmus MC, University Medical Center Rotterdam) for Cas9 protein synthesis, and John O’Donnell (MRC Laboratory of Molecular Biology) for help with thermal stability assays.

## Sources of Funding

This work was supported by the Dutch Heart Foundation (2014T007 to Dr van de Laar) and the Medical Research Council (MC_UP_A022_1007 to Dr Hegde). Dr van Ham and Dr van de Laar are supported by an Erasmus University Rotterdam Fellowship.

## Disclosures

None.

## Supplementary Material


