# Supplementary material for: Biallelic Variants in ASNA1, Encoding a Cytosolic Targeting Factor of Tail-Anchored Proteins, Cause Rapidly Progressive Pediatric Cardiomyopathy
Source: Circ Genom Precis Med. 2019 Aug 28;12(9):e002507. doi: 10.1161/CIRCGEN.119.002507 (PMC7205403; doi:10.1161/CIRCGEN.119.002507)
Supplement: Supplementary file 1 [file hcg-12-e002507-s001.pdf]

SUPPLEMENTAL MATERIAL

SUPPLEMENTAL METHODS …………………………………..………….………..………2

SUPPLEMENTAL TABLES

Table S1. List of primer sequences used for Sanger sequencing ………………………………..….8

Table S2. List of oligonucleotide sequences used in zebrafish studies …………………………..……9

Table S3. List of predicted human tail-anchored proteins ………………………………………...10

Table S4. Animal models for ASNA1-mediated TA protein insertion related genes ……………..….34

SUPPLEMENTAL FIGURES

Figure S1. CRISPR/Cas9-induced *asna1* deletion in zebrafish ……………………………...……36

Figure S2. Lateral views of the heart in wild-type and mutant zebrafish ………………...……….37

Figure S3. M-mode imaging in both patients ……………………………………………………..…38

Figure S4. Electrocardiography recordings of both patients ……………………………….…..….39

Figure S5. Recombinant expression and purification of ASNA1 from *E. coli* ……………...………40

SUPPLEMENTAL VIDEOS ………………………..…………...…………….……………42

SUPPLEMENTAL REFERENCES ………………..…………………………………………43

SUPPLEMENTAL METHODS

Clinical evaluation. The diagnosis of DCM was made based on current practice guidelines.[1, 2] Biochemical analysis in both affected siblings included quantitative analysis of lactate, amino acids, organic acids, carnitine and acylcarnitines, oligosaccharides, and isoelectric focusing of transferrin and apolipoprotein C-III. Family members who participated in this study underwent cardiac screening with electrocardiogram and echocardiography.

Exome sequencing. Genomic DNA was extracted from peripheral blood samples using standard protocols, and fragmented by sonication. Exons were captured using the SureSelect Human All Exon V4 (Agilent Technologies). Sequencing was performed on a Hiseq 2000 system (Illumina) for 101 base pair paired-end runs. Reads were mapped to the human reference genome GRCh37/hg19 using the Burrows-Wheeler Aligner (BWA).[3] Variants were called using the Genome Analysis Toolkit (GATK),[4] and filtered using Cartagenia Bench Lab software. We selected for rare variants, defined as variants with a minor allele frequency <0.1% in public variant databases, including the Genome Aggegregation Database (gnomAD), the NHLBI GO Exome Sequencing Project (ESP), and the Genome of the Netherlands (GoNL). We only included non-synonymous coding and splice site (±10 bp from exon-intron boundaries) variants with a minimum coverage of 10 reads. Apparent *de novo*, homozygous and compound heterozygous variants were considered for further analysis.

Sanger sequencing. Bidirectional Sanger sequencing of the entire coding region and exon-intron boundaries of the candidate genes was performed using PCR primers designed by Primer3 software (**Supplemental Table 1**). PCR products were purified and subsequently sequenced using the BigDye Terminator v3.1 kit on an ABI 3730xl DNA Analyzer (Life Technologies). Sequence data was analyzed using SeqScape v2.5 software. For annotation of DNA and protein changes, the mutation nomenclature recommendations from the Human Genome Variation Society were followed. To describe variants at the cDNA level, the A of the translation initiation codon of the reference sequence was designated as position +1.

Histology and immunostaining. Paraffin-embedded, hematoxylin and eosin (H&E) stained myocardial tissue from both affected siblings was examined using standard techniques. For immunohistochemistry, sections were deparaffinized and rehydrated before antibody retrieval. Primary antibodies included: full-length rabbit polyclonal anti-TRC40 (non-commercial, dilution 1:400),[5] mouse monoclonal anti-N-cadherin (Agilent Technologies #M3616, dilution 1:200), and mouse monoclonal anti-desmin (Ventana Medical Systems #760-2513, prediluted). The slides were counterstained with hematoxylin II for 8 minutes and bluing reagent for 8 minutes according to the manufacturer's instructions (Ventana Medical Systems). Immunostained preparations were analyzed by light microscopy. Glutaraldehyde-fixed myocardial tissue was prepared for electron microscopy. Immunolabeling was performed on cryosections as described previously.[6] Primary antibodies included: mouse monoclonal anti-N-cadherin (Sigma-Aldrich #C3865, 1:800) and mouse monoclonal anti-emerin (Novocastra Laboratories #NCL-EMERIN). Secondary labeling was performed with appropriate Texas Red (N-cadherin) or fluorescein isothiocyanate–conjugated antibodies (emerin). After immunolabeling, sections were analysed with a Nikon Eclipse 80i epifluorescence microscope, and images were taken using a DS-2BWc digital sight camera and NIS-Elements BR 3.0 software (Nikon Instruments).

Western blotting. Cultured skin fibroblasts from patient II:2 and three control individuals were lysed in 100 µL TNE buffer [50 mM Tris-HCl (pH 7.6), 100 mM NaCl, 50 mM NaF, 1% (v/v) Triton X-100] and cOmplete Protease Inhibitor Cocktail tablets (Roche Applied Science). Lysates were centrifuged for 10 minutes at 10,000 rpm to remove small cell debris. Equal amounts (20 µg or 40 µg) of protein were separated on a 4-15% precast polyacrylamide gel (Bio-Rad Laboratories). Rabbit polyclonal antibodies against the full-length (non-commercial; dilution 1:2000) and N-terminus (non-commercial; dilution 1:2000) of human ASNA1 were used for detection.[5] Results were normalized to the GAPDH loading control. Note: these experiments were only performed in patient II:2, as we did not have access to cultured skin fibroblasts from patient II:3.

***In vitro* synthesis of mRNA.** Total RNA was extracted from human skin fibroblasts using the RNeasy Mini Kit (QIAGEN), and converted into cDNA using the iScript Reverse Transcription

Supermix (Bio-Rad Laboratories, #1708840). Products were ligated into the pCMV6-entry vector with C-terminal Myc-DDK tag, and subsequently transformed into XL10-Gold ultracompetent cells (Stratagene). All constructs were verified by DNA sequencing. Expression of recombinant proteins was checked after transfection into human embryonic kidney (HEK) 293 cells using previously described rabbit polyclonal antibodies raised against the full-length and N-terminal peptide of human ASNA1.[5] Linearized constructs were used as a template for *in vitro* synthesis of capped mRNA using the mMESSAGE mMACHINE T7 Transcription Kit (Thermo Fisher Scientific).

Purification of recombinant ASNA1. The construct for wild-type zebrafish ASNA1 (TRC40) expression in *E. coli* has been described previously.[7] It contains an N-terminal 6xHis tag and tobacco etch virus (TEV) protease cleavage site, followed by the full-length ASNA1 open reading frame. The Val163Ala variant was introduced into this construct by site-directed mutagenesis and verified by sequencing. Expression and purification from *E. coli* used minor modifications of previously published methods.[7, 8] Briefly, the Rosetta BL21(DE3) pLysS strain of *E. coli* (Novagen) was transformed with the plasmid for wild-type or mutant ASNA1, and a single colony was used to grow an overnight starter culture. This was expanded to either 1 L or 6 L (for the wild-type and mutant cultures, respectively), and when the absorbance at 600 nm was between 0.4 to 0.6, isopropyl β-D-1-thiogalactopyranoside (IPTG) was added to 1 mM. After 3 hours at 37°C, the cells were collected by centrifugation at 4°C, washed once in ice cold PBS supplemented with 250 mM NaCl, and re-collected by centrifugation. The washed cells were resuspended in 35 mL of ice cold lysis buffer [PBS with 250 mM NaCl, 5 mM 2-mercaptoethanol, and 1X cOmplete EDTA-free Protease Inhibitor Cocktail mix (Roche)]. After lysis by sonication, the insoluble material was sedimented by centrifugation at 18,000 rpm for 30 minutes at 4°C. The soluble extract was adjusted to 20 mM imidazole, then passed over a 3 mL column of Ni-NTA resin, washed three times in 10 mL of lysis buffer supplemented with 20 mM imidazole, and eluted with lysis buffer supplemented with 250 mM imidazole. The peak fractions (identified by absorbance at 280 nm) were pooled, mixed with TEV protease (at a protein ratio of 1:100), and dialyzed overnight against dialysis buffer (150 mM KAc, 50 mM HEPES, pH 7.4, 2 mM MgCl2, 10% glycerol, 7 mM 2-mercaptoethanol). Insoluble material was

removed by centrifugation, and the dialyzed sample was passed over a 3 mL column of Ni-NTA to remove the cleaved tag and TEV protease. The flow-through was collected and concentrated to ~4 mg/mL by centrifugal concentrators (Amicon). The protein was snap-frozen in liquid nitrogen and stored in aliquots at -80°C.

Analysis of ASNA1 protein function *in vitro*. Thermal stability of the purified wild-type and Val163Ala mutant ASNA1 protein was analyzed using the Prometheus NT.48 system (NanoTemper Technologies). Purified protein at 0.8 mg/mL was monitored for intrinsic tryptophan fluorescence during a temperature ramp from 20°C to 95°C. A change in the ratio of emission at 330 nm and 350 nm was used to measure unfolding. The ability of ASNA1 protein to capture TA protein was assayed exactly as described previously.[7] In short, ^{35}S-labeled TA protein containing the transmembrane domain of VAMP2 (referred to simply as VAMP2 hereinafter) was assembled with the upstream chaperone SGTA. The TA protein contained a photo-crosslinking residue within the transmembrane domain to monitor its interactions. The SGTA-VAMP2 complex was then mixed with the bridging cBAG6 complex and either wild-type or mutant ASNA1. After incubating at 32°C for 90 seconds, the reaction was transferred to ice and irradiated with UV to induce crosslinking for 10 minutes. The samples were analyzed by SDS-PAGE and autoradiography to determine whether VAMP2 was successfully transferred from SGTA to ASNA1. To test the functionality of ASNA1 for TA protein insertion, a complex between ASNA1 and VAMP2 was assembled as before,[7] and incubated with ER microsomes for between 0 to 15 minutes at 32°C. The samples were then analyzed by SDS-PAGE and autoradiography. Insertion of the TA protein was monitored by its glycosylation at a site located near the C-terminus. The ER microsomes used for this assay were derived from HEK293 cells and were prepared as described before.[9]

CRISPR/Cas9 targeting of zebrafish *asna1*. Zebrafish *asna1* (ENSDARG00000018190) was targeted by Cas9/gRNA complex injection as described previously.[10] The online program CRISPRscan (www.crisprscan.org) was used to used to design a single-stranded guide RNA (gRNA) targeting exon 5 in *asna1* (5’-CCAAACTGGAGGAGACGCTGC-3’), approximately in between the variants identified in the parents. The gRNAs were obtained by *in vitro* transcription of synthetic

oligonucleotides containing a minimal T7 RNA polymerase promoter using the mMESSAGE mMACHINE T7 Ultra Kit (Thermo Fisher Scientific). SP-Cas9 plasmid was a gift from Niels Geijssen (Addgene plasmid #62731).[11] A mix of 100 pg of either gRNA and 650 pg Cas9 protein was injected into single-cell stage zebrafish embryos. Injected embryos were raised to adulthood (F0) and analyzed for genomic modifications at the target site by Sanger sequencing and the online tool Tracking Indel by DEcompensition (TIDE).[12] In two individual F0 founder fish, ~30% of the mapped reads contained indels at the target site in exon 5 (**Supplemental Figure 1**). We screened their offspring (F1) for germline transmission using PCR followed by restriction enzyme digestion (**Supplemental Table 2**), and identified three fish (25%) that carried a heterozygous 7 base pair deletion (Δ7). These fish were used for further breeding to create a stable mutant line. For rescue experiments, 300 pg wild-type or mutant human *ASNA1* mRNA (see above) was injected in the yolk at the single-cell stage. Expression of MYC-tagged human ASNA1 was confirmed by Western blot analysis with an anti-MYC primary antibody.

Phenotypic analysis of mutant zebrafish. Zebrafish were anesthetized with tricaine methanesulfonate (MS-222) and imaged using a Leica M165 FC stereo microscope connected to a Leica DFC550 digital camera. Zebrafish were positioned horizontally in 5% methylcellulose to obtain a lateral view of the ventricle (**Supplemental Figure 2**). Heart rate (beats/minute) was calculated by three independent counts of the number of beats in 15 second intervals. Fractional shortening (%) was derived from linear measurements of the ventricle at end-diastole and end-systole.[13] Blood flow rate, that can be used an indirect measure of cardiac function, was determined by visual inspection of circulating red blood cells passing through the dorsal aorta and classified as “normal”, “decreased” or “absent”. For microscopic analysis, zebrafish larvae (*n*=4 for each group) were anesthetized, fixed in Karnovsky fixative (PBS containing 2% paraformaldehyde and 3% glutaraldehyde), and embedded in Epon. Semithin sections (1 μm) were stained with toluidine blue and studied under a light microscope. Ultrathin sections (70 nm) were stained with 5% uranyl acetate and 2.5% lead citrate, and photographically recorded using a JEOL 1200-EX II transmission electron microscope.

Bioinformatics. In order to find proteins that might be affected by defective ASNA1-mediated membrane insertion, we obtained a list of all human single-pass membrane proteins from UniProt.[14] We first removed all proteins that contain an N-terminal signal sequence, and from the remainder, selected for proteins that contain a transmembrane domain within the last 50 residues from the C-terminus. The final list contained 286 predicted human TA proteins (**Supplemental Table 3**). We investigated the potential association between the corresponding genes and cardiomyopathy using the Online Mendelian Inheritance in Man database (https://www.omim.org).

Statistical analysis. Statistical analyses were performed using Microsoft Excel or GraphPad Prism software. Continuous variables were expressed as means ± standard deviation, and compared using the Student's *t*-test. Categorical variables were expressed as counts and percentages, and compared using the Fisher's exact test. An asterisk (*) indicates *p*-values lower than 0.05.

SUPPLEMENTAL TABLES

Supplemental Table 1. List of primer sequences used for Sanger sequencing.

**Target**	**Direction**	**Primer sequence (5' - 3')**	**Product size (bp)**
ASNA1 exon 1	F	tcctaaaaggcaagtaatgagga	367
	R	gtggaaaagccggtccttg	
ASNA1 exon 2	F	ctgctccagggaacctacc	389
	R	tggttcccttgtgagtatgttg	
ASNA1 exon 3	F	ccccttgtttttgacccttt	470
	R	AAGTTCATGCCCTTCACCAG	
ASNA1 exon 4	F	ATCGATGAGGCCATGAGCTA	375
	R	tgggaaggaaagggaattgt	
ASNA1 exon 5	F	ccactgggaggtatcaggag	599
	R	caggaggctagagggcagag	
ASNA1 exon 6	F	TCAAGGACCCTgtgagtgg	400
	R	caggaggctagagggcagag	
ASNA1 exon 7	F	cactctgtctctgccttcctg	299
	R	GGCTCCCCCTGTATTATGG	

F: forward; R: reverse.

Supplemental Table 2. List of oligonucleotide sequences used in zebrafish studies.

**Target**	**Direction**	**Primer sequence (5' - 3')**	**Product size (bp)**
asna1 exon 5	**F**	TAAAGCCCATTCCTGAGTGC	**404**
	**R**	TTGAAGTGGATGGATGATGG	

F: forward; R: reverse.

The PCR product was subjected to restriction enzyme digestion by Bsrl. As a result of the 7 bp deletion induced by CRISPR/Cas9, one Bsrl enzyme restriction site will be lost and the mutant allele will only be cut once. Subsequent gel electrophoresis will reveal three bands in wild-type (199, 131 and 74 bp), four bands in *asna1*$^{\Delta 7/+}$ (266, 199, 131 and 74 bp), and two bands in *asna1*$^{\Delta 7/\Delta 7}$ (266 and 131 bp) zebrafish.

Supplemental Table 3. List of predicted human tail-anchored proteins.

Entry	Protein names	TMD sequence
E0CX11	Short transmembrane mitochondrial protein 1	GFTLGNVVGMYLAQNYD
Q8NDB6	Protein FAM156A/FAM156B (Transmembrane protein 29/29B)	WETLVQGLSGLTLSLGT
Q9H7X2	Uncharacterized protein C1orf115	VVIGLQGFAAAYSAPFAVATSVV
Q8TCY0	Small integral membrane protein 11B	MEFPLCGCLSLILHHFA
Q96PS6	Putative uncharacterized protein GAFA-1 (Gene associated with FGF-2 activity protein 1)	IHLYVMASAMSSSPIFFFFQ
O75438	NADH dehydrogenase [ubiquinone] 1 beta subcomplex subunit 1 (Complex I-MNLL) (CI-MNLL) (NADH-ubiquinone oxidoreductase MNLL subunit)	HWVHVLVPMGFVIGCYL
Q9H1C7	Cysteine-rich and transmembrane domain-containing protein 1	LGPSTCLTACWTALCCCC
Q9HDD0	Phospholipid-metabolizing enzyme A-C1 (EC 2.3.1.-) (EC 3.1.1.-) (HRAS-like suppressor 1) (HRSL1)	ISTVEFVTAAVGVFSFLGLFPKGQ
L0R6Q1	SLC35A4 upstream open reading frame protein	ASAVLGFAVGTCTGIYAAQAYAV
Q96I36	Cytochrome c oxidase assembly protein COX14	FSTSMMLLTVYGGYLCSVRVYHY
P21397	Amine oxidase [flavin-containing] A (EC 1.4.3.4) (Monoamine oxidase type A) (MAO-A)	VSGLLKIIGFSTSVTALGFVL
O75452	Retinol dehydrogenase 16 (EC 1.1.-.-) (Microsomal NAD(+)-dependent retinol dehydrogenase 4) (RoDH-4) (Short chain dehydrogenase/reductase family 9C member 8) (Sterol/retinol dehydrogenase)	LLYLPMSYMPTFLVDAIMYWV
Q9BVW6	Small integral membrane protein 2	GHAISILFGFWTSFICDTYIVLA

Q75NE6	Putative microRNA 17 host gene protein (Putative microRNA host gene 1 protein)	LNVPKLVLIYLQSHFVLFFFSMC
Q9UMX3	Bcl-2-related ovarian killer protein (hBOK) (Bcl-2-like protein 9) (Bcl2-L-9)	WLVAALCSFGRFLKAAFFVLL
Q8TCP9	Protein FAM200A	ILLLLPFTTTYLCELGFSIL
O95167	NADH dehydrogenase [ubiquinone] 1 alpha subcomplex subunit 3 (Complex I-B9) (CI-B9) (NADH-ubiquinone oxidoreductase B9 subunit)	LVVSFVVGGLAVILPPLSPYF
Q07812	Apoptosis regulator BAX (Bcl-2-like protein 4) (Bcl2-L-4)	TVTIFVAGVLTASLTIWKKMG
Q8WXE9	Stonin-2 (Stoned B)	IWLMLPTPFVHPTTLPLLFLLAM
Q9NX95	Syntabulin (Golgi-localized syntaphilin-related protein) (Syntaxin-1-binding protein)	SFLVDLLAVAAPVVPTVLWAF
Q6ZSY5	Protein phosphatase 1 regulatory subunit 3F (R3F)	VLAGLVVVPVALNSGVSLLVL
Q3KP22	Membrane-anchored junction protein	AATGFFGFLSSLFPFRYFF
A8MTT3	Protein CEBPZOS (CEBPZ antisense RNA 1) (CEBPZ opposite strand)	GVLVAELVGVFGAYFLFS
Q07817	Bcl-2-like protein 1 (Bcl2-L-1) (Apoptosis regulator Bcl-X)	FNRWFLTGMTVAGVVLL
P56378	6.8 kDa mitochondrial proteolipid	VYQEIWIGMGLMGFIVYKI
O15079	Syntaphilin	YIVDLLAVVVPAVPTVAWLC
Q9NRY6	Phospholipid scramblase 3 (PL scramblase 3) (Ca(2+)-dependent phospholipid scramblase 3)	VKAVLLGATFLIDYMFF
Q9NUB4	Uncharacterized protein C20orf141	LLLLMGLGPLLRACGMPLTLL
O95139	NADH dehydrogenase [ubiquinone] 1 beta subcomplex subunit 6 (Complex I-B17) (CI-B17) (NADH-	SIFVFTHVLVPVWIIHYYM

	ubiquinone oxidoreductase B17 subunit)	
O43676	NADH dehydrogenase [ubiquinone] 1 beta subcomplex subunit 3 (Complex I-B12) (CI-B12) (NADH-ubiquinone oxidoreductase B12 subunit)	VFFKGFKWGFAAFVVAVGAEYYL
Q8NCU8	Uncharacterized protein encoded by LINC00116	LQLSVLVAFASGVLLGW
Q8N4H5	Mitochondrial import receptor subunit TOM5 homolog	SIRNFLIYVALLRVTPFIL
Q9NRY7	Phospholipid scramblase 2 (PL scramblase 2) (Ca(2+)-dependent phospholipid scramblase 2)	MKAVMIGACFLIDYMFF
Q8N7S6	Uncharacterized protein ARIH2OS (Ariadne-2 homolog opposite strand protein)	CILTALLAVSFHSIGVVIMTS
Q9UL19	Retinoic acid receptor responder protein 3 (EC 3.1.1.-) (HRAS-like suppressor 4) (HRSL4) (RAR-responsive protein TIG3) (Retinoid-inducible gene 1 protein) (Tazarotene-induced gene 3 protein)	KVEVGVATALGILVVAGCSFAI
A2RU48	Single-pass membrane and coiled-coil domain-containing protein 3	IGASLLGSIGVAVLGLGIDMI
P03928	ATP synthase protein 8 (A6L) (F-ATPase subunit 8)	VWPTMITPMLLTLFLIT
P0DJ07	Protein PET100 homolog, mitochondrial	IFRMIIYLTFPVAMFWVS
Q5TGZ0	MICOS complex subunit MIC10 (Mitochondrial inner membrane organizing system protein 1)	AVVKIGTGFGLGIVFSLTFF
O15162	Phospholipid scramblase 1 (PL scramblase 1) (Ca(2+)-dependent phospholipid scramblase 1) (Erythrocyte phospholipid scramblase) (MmTRA1b)	MKAVMIGACFLIDFMFF
O15239	NADH dehydrogenase [ubiquinone] 1 alpha subcomplex subunit 1 (Complex I-MWFE) (CI-MWFE) (NADH-ubiquinone oxidoreductase MWFE subunit)	MWFEILPGLSVMGVCLLIPGL

A6NCI5	Putative transmembrane protein encoded by LINC00862 (Small integral membrane protein 16)	IMALILMPSLHCFGNILILLF
Q9NRQ2	Phospholipid scramblase 4 (PL scramblase 4) (Ca(2+)-dependent phospholipid scramblase 4) (Cell growth-inhibiting gene 43 protein) (TRA1)	MKAMIFGACFLIDFMYF
Q9BSJ5	Uncharacterized protein C17orf80 (Cell migration-inducing gene 3 protein) (Human lung cancer oncogene 8 protein) (HLC-8)	GFGGITMLFTGYFVLCCSWSF
P08574	Cytochrome c1, heme protein, mitochondrial (Complex III subunit 4) (Complex III subunit IV) (Cytochrome b-c1 complex subunit 4) (Ubiquinol-cytochrome-c reductase complex cytochrome c1 subunit) (Cytochrome c-1)	MLMMMALLVPLVYTI
Q9HD87	Putative uncharacterized protein C6orf50 (Nasopharyngeal carcinoma-associated gene 19 protein)	IISLLAIFIKMCLWLWKQFL
P60602	Reactive oxygen species modulator 1 (ROS modulator 1) (Epididymis tissue protein Li 175) (Glyrichin) (Mitochondrial targeting GxxxG motif protein) (MTGM) (Protein MGR2 homolog)	GFVMGCAVGMAAGALFGTFSCLR
Q9P0U1	Mitochondrial import receptor subunit TOM7 homolog (Translocase of outer membrane 7 kDa subunit homolog)	FAIRWGFIPLVIYLGF
P0DMW3	Small integral membrane protein 10-like protein 1	FFYFYILASVILNVHLQVY
Q96HG1	Small integral membrane protein 10	FFYFYILASVILNVHLQVY
Q96IX5	Up-regulated during skeletal muscle growth protein 5 (Diabetes-associated protein in insulin-sensitive tissues) (HCV F-transactivated protein 2)	TLTGRMNCVLATYGSIALIVLYF

O95237	Lecithin retinol acyltransferase (EC 2.3.1.135) (Phosphatidylcholine--retinol O-acyltransferase)	VLASAVLGLASIVCTGLVSYT
E9PQ53	NADH dehydrogenase [ubiquinone] 1 subunit C2, isoform 2 (NDUFC2-KCTD14 readthrough transcript protein)	GLHRQLLYITAFFFAGYYLV
O95298	NADH dehydrogenase [ubiquinone] 1 subunit C2 (Complex I-B14.5b) (CI-B14.5b) (Human lung cancer oncogene 1 protein) (HLC-1) (NADH-ubiquinone oxidoreductase subunit B14.5b)	GLHRQLLYITAFFFAGYYLV
P56134	ATP synthase subunit f, mitochondrial	ISGITMVLACYVLFSYSFSY
A0A5B9	T-cell receptor beta-2 chain C region	TILYEILLGKATLYAVLVSALVL
P53816	HRAS-like suppressor 3 (HRSL3) (EC 3.1.1.32) (EC 3.1.1.4) (Adipose-specific phospholipase A2) (AdPLA) (Group XVI phospholipase A1/A2) (H-rev 107 protein homolog) (H-REV107) (HREV107-1) (HRAS-like suppressor 1) (HREV107-3) (Renal carcinoma antigen NY-REN-65)	VIIAASVAGMGLAAMSLIGVMFS
Q7Z412	Peroxisome assembly protein 26 (Peroxin-26)	FFSLPFKKSLLAALILCLLVV
Q9NS69	Mitochondrial import receptor subunit TOM22 homolog (hTom22) (1C9-2) (Translocase of outer membrane 22 kDa subunit homolog)	ALWIGTTSFMILVLPVVFET
Q9GZY8	Mitochondrial fission factor	VMYSITVAFWLLNSWLWF
Q13505	Metaxin-1 (Mitochondrial outer membrane import complex protein 1)	ILSVLAGLAAMVGYALLSGIV
P01848	T-cell receptor alpha chain C region	VIGFRILLLKVAGFNLLMTL
P27338	Amine oxidase [flavin-containing] B (EC 1.4.3.4) (Monoamine oxidase type B) (MAO-B)	PGLLRLIGLTTIFSATALGFLAHKRGL

O00198	Activator of apoptosis harakiri (BH3-interacting domain-containing protein 3) (Neuronal death protein DP5)	WPWLCAAAQVAALAAWLLG
B7Z8K6	T-cell receptor delta chain C region	LGLRMLFAKTVAVNFLLTAKLFF
O95168	NADH dehydrogenase [ubiquinone] 1 beta subcomplex subunit 4 (Complex I-B15) (CI-B15) (NADH-ubiquinone oxidoreductase B15 subunit)	LMGALCGFGPLIFIYYII
Q16611	Bcl-2 homologous antagonist/killer (Apoptosis regulator BAK) (Bcl-2-like protein 7) (Bcl2-L-7)	ILNVLVVLGVVLLGQFVV
O60238	BCL2/adenovirus E1B 19 kDa protein-interacting protein 3-like (Adenovirus E1B19K-binding protein B5) (BCL2/adenovirus E1B 19 kDa protein-interacting protein 3A) (NIP3-like protein X) (NIP3L)	VFIPSLFLSHVLALGLGIYIG
Q96N68	Putative uncharacterized protein C18orf15	MCVCVHVCACVYVCMCVLVCM
Q07820	Induced myeloid leukemia cell differentiation protein Mcl-1 (Bcl-2-like protein 3) (Bcl2-L-3) (Bcl-2-related protein EAT/mcl1) (mcl1/EAT)	IRNVLLAFAGVAGVGAGLAYL
Q09013	Myotonin-protein kinase (MT-PK) (EC 2.7.11.1) (DM-kinase) (DMK) (DM1 protein kinase) (DMPK) (Myotonic dystrophy protein kinase)	LLLFAVVLSRAAALGCIGLVA
Q9Y3D6	Mitochondrial fission 1 protein (FIS1 homolog) (hFis1) (Tetratricopeptide repeat protein 11) (TPR repeat protein 11)	LVGMAIVGGMALGVAGLAGLI
Q96JJ6	Junctophilin-4 (JP-4) (Junctophilin-like 1 protein)	LVVGAVALLDLSLAFLFSQLLT
Q96K12	Fatty acyl-CoA reductase 2 (EC 1.2.1.84) (Male sterility domain-containing protein 1)	NIHYLFNTALFLIAWRLLIA

Q9H0X9	Oxysterol-binding protein-related protein 5 (ORP-5) (OSBP-related protein 5) (Oxysterol-binding protein homolog 1)	SWFLLCVFLACQLFINHIL
F7VJQ1	Alternative prion protein (AltPrP)	WWWLGAASWWWLGAAPWWWLG
Q96KF7	Small integral membrane protein 8	PVMAFGLVTLSLCVAYIGYLHAI
Q8WVI0	Small integral membrane protein 4	FGIYRFLPFFFVLGGTMEWIMI
P37268	Squalene synthase (SQS) (SS) (EC 2.5.1.21) (FPP:FPP farnesyltransferase) (Farnesyl-diphosphate farnesyltransferase)	PIYLSFVMLLAALSWQYLTTL
Q3B7S5	Small integral membrane protein 21	HIRFFTLLVLFHVMVLL
P10415	Apoptosis regulator Bcl-2	FSWLSLKTLLSLALVGACITLG
H7C350	Coiled-coil domain-containing protein 188	LLLGALLVWTAAYVYVV
Q14318	Peptidyl-prolyl cis-trans isomerase FKBP8 (PPIase FKBP8) (EC 5.2.1.8) (38 kDa FK506-binding protein) (38 kDa FKBP) (FKBP-38) (hFKBP38) (FK506-binding protein 8) (FKBP-8) (FKBPR38) (Rotamase)	WLFGATAVALGGVALSVVIAA
Q8IVJ8	AP20 region protein 1	IALALAGPGAILILELSWFLG
P0DMT0	Myoregulin	VGRLLKILFVIFVDLISIIYV
Q8N326	Uncharacterized protein C10orf111	MSLLLLPAFSGLTWAPFLFLF
P60059	Protein transport protein Sec61 subunit gamma	FQKIAMATAIGFAIMGFIGFFVKLIHIPI

Q12983	BCL2/adenovirus E1B 19 kDa protein-interacting protein 3	VFLPSLLLSHLLAIGLGIYIG
O43677	NADH dehydrogenase [ubiquinone] 1 subunit C1, mitochondrial (Complex I-KFYI) (CI-KFYI) (NADH-ubiquinone oxidoreductase KFYI subunit)	WLKVGFTLGTTVFLWIYLI
Q8N2K1	Ubiquitin-conjugating enzyme E2 J2 (EC 2.3.2.23) (E2 ubiquitin-conjugating enzyme J2) (Non-canonical ubiquitin-conjugating enzyme 2) (NCUBE-2)	GLLGGALANLFVIVGFAAFAY
O96011	Peroxisomal membrane protein 11B (Peroxin-11B) (Peroxisomal biogenesis factor 11B) (Protein PEX11 homolog beta) (PEX11-beta)	GIVGLCGLVSSILSILTLIYPWL
Q86T96	E3 ubiquitin-protein ligase RNF180 (EC 2.3.2.27) (RING finger protein 180) (RING-type E3 ubiquitin transferase RNF180)	MVIIYIYSVNWVIGFIVFCFL
Q5T8D3	Acyl-CoA-binding domain-containing protein 5	GVLTFAIIWPFIAQWLVYLYY
Q9H4I9	Essential MCU regulator, mitochondrial (Single-pass membrane protein with aspartate-rich tail 1, mitochondrial)	FGLLRVFSIVIPFLYVGTLI
Q8NA58	Poly(A)-specific ribonuclease PNLDC1 (EC 3.1.13.4) (PARN-like domain-containing protein 1) (Poly(A)-specific ribonuclease domain-containing protein 1) (HsPNLDC1)	VNCLLQVCGIVTAWALLAFIL
O95169	NADH dehydrogenase [ubiquinone] 1 beta subcomplex subunit 8, mitochondrial (Complex I-ASHI) (CI-ASHI) (NADH-ubiquinone oxidoreductase ASHI subunit)	LFGFLAFMIFMCWVGDVYPVY
O94966	Ubiquitin carboxyl-terminal hydrolase 19 (EC 3.4.19.12) (Deubiquitinating enzyme 19) (Ubiquitin	FVLGTVAALVALVLNVFYPLV

	thioesterase 19) (Ubiquitin-specific-processing protease 19) (Zinc finger MYND domain-containing protein 9)	
P01850	T-cell receptor beta-1 chain C region	ILLGKATLYAVLVSALVLMAM
Q96A26	Protein FAM162A (E2-induced gene 5 protein) (Growth and transformation-dependent protein) (HGTD-P)	ISYLMIALTVVGCIFMVI
Q969F0	Fetal and adult testis-expressed transcript protein (Cancer/testis antigen 43) (CT43) (Tumor antigen BJ-HCC-2)	TLIIAVLVSASIANLWLWM
Q8N5G0	Small integral membrane protein 20 (Mitochondrial translation regulation assembly intermediate of cytochrome c oxidase protein of 7 kDa) (MITRAC7)	TALIFGGFISLIGAAFYPIYF
Q86UQ5	Gilles de la Tourette syndrome chromosomal region candidate gene 1 protein	AICMEVFLFLWFIAPIYACVC
P00167	Cytochrome b5 (Microsomal cytochrome b5 type A) (MCB5)	WWTNWVIPAISAVAVALMYRLYM
Q9NPU4	Uncharacterized protein C14orf132	AVLLWIAIIATLGNIVVVGVV
P60468	Protein transport protein Sec61 subunit beta	VPVLVMSLLFIASVFMLHIWG
Q9NWW9	HRAS-like suppressor 2 (EC 2.3.1.-) (EC 3.1.1.-)	AVTTVGVAAGLLAAASLVGILLA
Q7Z3B0	Small integral membrane protein 15	YGFLTTVILALTPLFLASAVL
Q9Y5L2	Hypoxia-inducible lipid droplet-associated protein (Hypoxia-inducible gene 2 protein)	LYLLGVVLTLLSIFVRV
Q96FB5	Protein RRNAD1 (Ribosomal RNA adenine dimethylase domain-containing protein 1)	VVAFFSLALLLAPLVETLILL

B2RUZ4	Small integral membrane protein 1 (Vel blood group antigen)	LGIAMKVLGGVALFWIIFILG
Q9Y2R0	Cytochrome c oxidase assembly factor 3 homolog, mitochondrial (Coiled-coil domain-containing protein 56) (Mitochondrial translation regulation assembly intermediate of cytochrome c oxidase protein of 12 kDa)	IVTGLGIGALVLAIYGYTFYS
Q8IXI1	Mitochondrial Rho GTPase 2 (MIRO-2) (hMiro-2) (EC 3.6.5.-) (Ras homolog gene family member T2)	GLLGVVGAAVAAVLSFSLYRVLV
P0C6T2	Dolichyl-diphosphooligosaccharide--protein glycosyltransferase subunit 4	VQLAIFANMLGVSLFLLVVLY
Q14BN4	Sarcolemmal membrane-associated protein (Sarcolemmal-associated protein)	WMPMLAALVAVTAIVLYVPGL
Q8WVX9	Fatty acyl-CoA reductase 1 (EC 1.2.1.84) (Male sterility domain-containing protein 2)	IRYGFNTILVILIWRIFI
Q14D33	Receptor-transporting protein 5 (3CxxC-type zinc finger protein 5) (CXXC-type zinc finger protein 11)	FWIWVSMTVCVFWLMCM
Q8NI28	Putative uncharacterized protein encoded by LINC01006 (Long intergenic non-protein coding RNA 1006)	WIPLLLVAGCVSCFVGLAVCV
I3L1I5	Putative uncharacterized protein LOC100996504	VLSIILLGSLLMCASSFCFAL
A4D256	Dual specificity protein phosphatase CDC14C (EC 3.1.3.16) (EC 3.1.3.48) (CDC14 cell division cycle 14 homolog C)	ILLPSPLAVLTFTLCSVVIWWIV
Q9Y385	Ubiquitin-conjugating enzyme E2 J1 (EC 2.3.2.23) (E2 ubiquitin-conjugating enzyme J1) (Non-canonical ubiquitin-conjugating enzyme 1) (NCUBE-1) (Yeast ubiquitin-conjugating enzyme UBC6 homolog E) (HsUBC6e)	DHGGSAVLIVILTLALAALIF

Q6ZS62	Colorectal cancer-associated protein 1	LYGCFCVGLVSGMAISVLLLA
A6NFE2	Single-pass membrane and coiled-coil domain-containing protein 2	IFIMFDVLTVTGLLCYILFFG
Q9NS64	Protein reprimo	VVQIAVMCVLSLTVVFGIFFL
Q16821	Protein phosphatase 1 regulatory subunit 3A (Protein phosphatase 1 glycogen-associated regulatory subunit) (Protein phosphatase type-1 glycogen targeting subunit) (RG1)	YFLLFLIFLITVYHYDLMIGL
Q9NQG1	Protein MANBAL	YGLFLGAIFQLICVLAIIVPI
P58511	Small integral membrane protein 11A	PLLLYILAAKTLILCLTFAGVKM
Q9Y228	TRAF3-interacting JNK-activating modulator (TRAF3-interacting protein 3)	WLPVLMVVIAAALAVFLA
Q9BXU9	Calcium-binding protein 8 (CaBP8) (Calneuron I) (Calneuron-1)	LICAFAMAFIISVMLIAANQI
A6NL05	Protein FAM74A7	LSLLLHLAVFLWIIIAINFSN
Q4VXF1	Putative protein FAM74A3	LSLLLHLAVFLWIIIAINFSN
Q5RGS3	Protein FAM74A1	LSLLLHLAVFLWIIIAINFSN
Q5T6X4	Protein FAM162B	VKACYIMIGLTIIACFAVIVS
Q9NXE4	Sphingomyelin phosphodiesterase 4 (EC 3.1.4.12) (Neutral sphingomyelinase 3) (nSMase-3) (nSMase3) (Neutral sphingomyelinase III)	LLLAFFVASLFCVGPLPCTLL
P51648	Fatty aldehyde dehydrogenase (EC 1.2.1.3) (Aldehyde dehydrogenase 10) (Aldehyde dehydrogenase family 3 member A2) (Microsomal aldehyde dehydrogenase)	LGLLLLTFLGIVAAVLV

Q8IXI2	Mitochondrial Rho GTPase 1 (MIRO-1) (hMiro-1) (EC 3.6.5.-) (Rac-GTP-binding protein-like protein) (Ras homolog gene family member T1)	WLRASFGATVFAVLGFAMYKALL
Q8N4K4	Reprimo-like protein	VAQIAVLCVLSLTVVFGVFFL
Q8N6R1	Stress-associated endoplasmic reticulum protein 2 (Ribosome-associated membrane protein RAMP4-2)	GPWLLALFVFVVCGSAIFQII
P58549	FXYD domain-containing ion transport regulator 7	TVQTVGMTLATILFLLGILIVIS
Q86V35	Calcium-binding protein 7 (CaBP7) (Calneuron II) (Calneuron-2)	LICAFAIAFIISVMLIAANQV
Q6ZNB6	NF-X1-type zinc finger protein NFXL1 (Ovarian zinc finger protein) (hOZFP)	YYLISVCGVVVVVFAWYI
O75056	Syndecan-3 (SYND3)	AVIVGGVVGALFAAFLVTLLI
P03986	T-cell receptor gamma-2 chain C region (T-cell receptor gamma chain C region PT-gamma-1/2)	MYLLLLLLKSVVYFAIITCCLL
A1L1A6	Immunoglobulin superfamily member 23	LLAAGILGAGALIAGMCFIII
Q8N5Y8	Mono [ADP-ribose] polymerase PARP16 (EC 2.4.2.30) (ADP-ribosyltransferase diphtheria toxin-like 15) (Poly [ADP-ribose] polymerase 16) (PARP-16)	SHWFTVMISLYLLLLLIVSVI
P61266	Syntaxin-1B (Syntaxin-1B1) (Syntaxin-1B2)	IMIIICCVVLGVVLASSIGGTLGL
Q9BZF1	Oxysterol-binding protein-related protein 8 (ORP-8) (OSBP-related protein 8)	YFIIFLLILLQVIINFMF
A6NGB0	Transmembrane protein 191C	VLGALQVLLTLPLLFLGLSLL
P0C7N4	Transmembrane protein 191B	VLGALQVLLTLPLLFLGLSLL
Q7Z419	E3 ubiquitin-protein ligase RNF144B (EC 2.3.2.-) (IBR domain-containing protein 2) (RING finger	VVGILVGLGIIALVTSPLLLL

	protein 144B) (p53-inducible RING finger protein)	
Q9UPX6	UPF0258 protein KIAA1024	IAALIAAAACTVILVIVVPIC
P54710	Sodium/potassium-transporting ATPase subunit gamma (Na(+)/K(+) ATPase subunit gamma) (FXYD domain-containing ion transport regulator 2) (Sodium pump gamma chain)	GGLIFAGLAFIVGLLILL
Q96LL3	Uncharacterized protein C16orf92	PGLFHHILVGLLVVAFFFLLF
Q9Y6X1	Stress-associated endoplasmic reticulum protein 1 (Ribosome-attached membrane protein 4)	GPWLLALFIFVVCGSAIFQII
Q16623	Syntaxin-1A (Neuron-specific antigen HPC-1)	IMIIICCVILGIVIASTVGGI
P60509	Endogenous retrovirus group PABLB member 1 Env polyprotein (Endogenous retrovirus group PABLB member 1) (Envelope polyprotein) (HERV-R(b) Env protein) (HERV-R(b)_3p24.3 provirus ancestral Env polyprotein) [Includes: Surface protein domain (SU); Transmembrane protein domain (TM)]	ILIVLATLWSVGIALCCGLYF
P50876	E3 ubiquitin-protein ligase RNF144A (EC 2.3.2.-) (RING finger protein 144A) (UbcM4-interacting protein 4) (Ubiquitin-conjugating enzyme 7-interacting protein 4)	VVGIFAGFGLLLLVASPFLLL
Q96DX8	Receptor-transporting protein 4 (28 kDa interferon-responsive protein) (3CxxC-type zinc finger protein 4)	PLNICVFILLLVFIVVKCFTS
Q12846	Syntaxin-4 (Renal carcinoma antigen NY-REN-31)	IAICVSITVVLLAVIIGVTVV
Q9UEU0	Vesicle transport through interaction with t-SNAREs homolog 1B (Vesicle transport v-SNARE protein	LSIIILLELAILGGLVYYKFF

	Vti1-like 1) (Vti1-rp1)	
A8MYB1	Transmembrane and coiled-coil domain-containing protein 5B	YFQYLTFMVLVFIRLLAYVIFHL
Q9BXK5	Bcl-2-like protein 13 (Bcl2-L-13) (Bcl-rambo) (Protein Mil1)	ILLFGGAAAVAILAVAIGVAL
Q6PJW8	Consortin	CILLVLLCIATVFLSVGGTAL
H3BV60	Transforming growth factor-beta receptor type 3-like protein (TGF-beta receptor type-3-like protein) (TGFR-3L) (Transforming growth factor-beta receptor type III-like protein) (TGF-beta receptor type III-like protein)	VVALVLAAFVLGAALAAGLGL
P17706	Tyrosine-protein phosphatase non-receptor type 2 (EC 3.1.3.48) (T-cell protein-tyrosine phosphatase) (TCPTP)	ILTKMGFMSVILVGAFVGWTLFF
Q8N111	Cell cycle exit and neuronal differentiation protein 1 (BM88 antigen)	LVAGGVAVAAIALILGVAFLV
E7ERA6	RING finger protein 223	LVSALLLMLFCVALWPVQCAL
O14653	Golgi SNAP receptor complex member 2 (27 kDa Golgi SNARE protein) (Membrin)	YFMIGGMLLTCVVMFLVVQYL
Q9BZ97	Putative transcript Y 13 protein	LLGWDLNLSLFLGLCLMLLLA
Q9P0L0	Vesicle-associated membrane protein-associated protein A (VAMP-A) (VAMP-associated protein A) (VAP-A) (33 kDa VAMP-associated protein) (VAP-33)	LPSLLVVIAAIFIGFFLGKFI
Q8N8J7	Uncharacterized protein C4orf32	VIVIFFWVMLWFLGLQALGLV
P37287	Phosphatidylinositol N-acetylglucosaminyltransferase subunit A (EC 2.4.1.198) (GlcNAc-PI synthesis	PVTGYIFALLAVFNFLFLIFL

	protein) (Phosphatidylinositol-glycan biosynthesis class A protein) (PIG-A)	
Q5VV42	Threonylcarbamoyladenosine tRNA methylthiotransferase (EC 2.8.4.5) (CDK5 regulatory subunit-associated protein 1-like 1) (tRNA-t(6)A37 methylthiotransferase)	CALRMSVGLALLGLLFAFFVKVY
Q8WWG1	Pro-neuregulin-4, membrane-bound isoform (Pro-NRG4) [Cleaved into: Neuregulin-4 (NRG-4)]	EAFVALAVLVTLIIGAFYFLC
Q96NA8	t-SNARE domain-containing protein 1	CFLSAGVTALLVIIIIIATSV
Q68G75	LEM domain-containing protein 1 (Cancer/testis antigen 50) (CT50) (LEM domain protein 1) (LEMP-1)	FPVGLKLAVLGIFIIVVFVYL
P0CF51	T-cell receptor gamma chain C region 1	YYMYLLLLLKSVVYFAIITCCLL
Q9NX14	NADH dehydrogenase [ubiquinone] 1 beta subcomplex subunit 11, mitochondrial (Complex I-ESSS) (CI-ESSS) (NADH-ubiquinone oxidoreductase ESSS subunit) (Neuronal protein 17.3) (Np17.3) (p17.3)	LVFFFGVSIILVLGSTFVAYL
Q8IUY3	GRAM domain-containing protein 2A	LLKVFFVLICFLVMSSSYLAF
Q9UNK0	Syntaxin-8	MIMVILLLLVAIVVVAV
Q9GZT6	Coiled-coil domain-containing protein 90B, mitochondrial	TIRYLAASVFTCLAIALGFYRFW
O95249	Golgi SNAP receptor complex member 1 (28 kDa Golgi SNARE protein) (28 kDa cis-Golgi SNARE p28) (GOS-28)	SLILGGVIGICTILLLLYAFH
Q9P0B6	Coiled-coil domain-containing protein 167	MLLSVAIFILLTLVYAYW

Q9HDC5	Junctophilin-1 (JP-1) (Junctophilin type 1)	IMIVLVMLLNIGLAILFVHFL
Q8NF91	Nesprin-1 (Enaptin) (KASH domain-containing protein 1) (KASH1) (Myocyte nuclear envelope protein 1) (Myne-1) (Nuclear envelope spectrin repeat protein 1) (Synaptic nuclear envelope protein 1) (Syne-1)	AALPLQLLLLLLIGLACLVPM
Q8TBA6	Golgin subfamily A member 5 (Cell proliferation-inducing gene 31 protein) (Golgin-84) (Protein Ret-II) (RET-fused gene 5 protein)	VFVIIYMALLHLWVMIVLLTY
Q53EP0	Fibronectin type III domain-containing protein 3B (Factor for adipocyte differentiation 104) (HCV NS5A-binding protein 37)	IIVLGFATLSILFAFILQYFL
Q13323	Bcl-2-interacting killer (Apoptosis inducer NBK) (BIP1) (BP4)	VLLALLLLLALLLPLLSGGLH
Q9Y6F6	Protein MRVI1 (Inositol 1,4,5-trisphosphate receptor-associated cGMP kinase substrate) (JAW1-related protein MRVI1)	WQVIWMMAAVMLVLTVVLGLY
Q8N912	Nutritionally-regulated adipose and cardiac enriched protein homolog	GGSLLLQLCVCVLLVLALGLY
Q13948	Protein CASP	IGFFYTLFLHCLVFLVLYKLA
Q8WXI7	Mucin-16 (MUC-16) (Ovarian cancer-related tumor marker CA125) (CA-125) (Ovarian carcinoma antigen CA125)	FWAVILIGLAGLLGVITCLIC
Q14789	Golgin subfamily B member 1 (372 kDa Golgi complex-associated protein) (GCP372) (Giantin) (Macrogolgin)	VPLLAAIYFLMIHVLLILCFT

Q8WXH2	Junctophilin-3 (JP-3) (Junctophilin type 3) (Trinucleotide repeat-containing gene 22 protein)	LVVMVILLNIGVAILFINFFI
Q8TC41	Probable E3 ubiquitin-protein ligase RNF217 (EC 2.3.2.-) (IBR domain-containing protein 1) (RING finger protein 217)	LIMVLGLALGAIAVVIGLFVF
Q9Y6H6	Potassium voltage-gated channel subfamily E member 3 (MinK-related peptide 2) (Minimum potassium ion channel-related peptide 2) (Potassium channel subunit beta MiRP2)	YMYILFVMFLFAVTVGSLILG
Q8NCQ3	Putative uncharacterized protein encoded by LINC00301	SFGLAIIGILLIACEIILFLT
P0DN84	Sarcoplasmic/endoplasmic reticulum calcium ATPase regulator DWORF (SERCA regulator DWORF) (Dwarf open reading frame) (DWORF)	VPILLLIGWIVGCIIMIYVVF
P0DL12	Small integral membrane protein 17	IVLVVCVLFLFLVLTGMPMMF
Q86Z14	Beta-klotho (BKL) (BetaKlotho) (Klotho beta-like protein)	LIFLGCCFFSTLVLLLSIAIF
P59025	Receptor-transporting protein 1 (3CxxC-type zinc finger protein 1)	IPWCLFWATVLLLIIYLQFSF
Q5QGT7	Receptor-transporting protein 2 (3CxxC-type zinc finger protein 2)	LSLRWCLFWASLCLLVVYLQFSF
Q6ZS82	Regulator of G-protein signaling 9-binding protein (RGS9-anchoring protein)	ALAAILFGAVLLAAVALAVCV
Q9NYM9	BET1-like protein (Golgi SNARE with a size of 15 kDa) (GOS-15) (GS15) (Vesicle transport protein GOS15)	LLCGMAVGLIVAFFILSYFLS
Q9NRQ5	Single-pass membrane and coiled-coil domain-containing protein 4 (Protein FN5)	TVVLPTLAVVVLLIVVFVYVA
Q0VAQ4	Small cell adhesion glycoprotein (Small transmembrane and glycosylated protein)	IAVVITVVFLTLLSVVILIFF

Q96AG4	Leucine-rich repeat-containing protein 59 (Ribosome-binding protein p34) (p34) [Cleaved into: Leucine-rich repeat-containing protein 59, N-terminally processed]	WAVLKLLLLLLLFGVAGGLVA
Q9BR39	Junctophilin-2 (JP-2) (Junctophilin type 2)	ILICMVILLNIGLAILFVHLL
P23763	Vesicle-associated membrane protein 1 (VAMP-1) (Synaptobrevin-1)	MMIMLGAICAIIVVVIVIYF
Q96JN2	Coiled-coil domain-containing protein 136 (Nasopharyngeal carcinoma-associated gene 6 protein)	IFSLPLVGLVVISALLWCWWA
P51809	Vesicle-associated membrane protein 7 (VAMP-7) (Synaptobrevin-like protein 1) (Tetanus-insensitive VAMP) (Ti-VAMP)	LTIIIIIVSIVFIYIIVSPLC
Q9BQQ7	Receptor-transporting protein 3 (3CxxC-type zinc finger protein 3) (Transmembrane protein 7)	SIFCCCVILIVIVVIVVKTAI
O00631	Sarcolipin	LFLNFTIVLITVILMWLLV
A6NCQ9	RING finger protein 222	LITLIAVVAVVAAILPWVLLV
Q8WWP7	GTPase IMAP family member 1 (Immunity-associated protein 1) (hIMAP1)	SWRLGLALLLGGALLFWVLL
Q96D05	Uncharacterized protein C10orf35	ILLLFLLMMLGVRGLLLVGLV
Q9Y2H6	Fibronectin type-III domain-containing protein 3A (Human gene expressed in odontoblasts)	ILVLFAFFSILIAFIIQYFVI
P04921	Glycophorin-C (Glycoconnectin) (Glycophorin-D) (GPD) (Glycoprotein beta) (PAS-2') (Sialoglycoprotein D) (CD antigen CD236)	DIVVIAGVIAAVAIVLVSLLFVML
Q8N8N0	E3 ubiquitin-protein ligase RNF152 (EC 2.3.2.27) (RING finger protein 152) (RING-type E3 ubiquitin transferase RNF152)	SGVCTVILVACVLVFLLGIVL

A6NNC1	Putative POM121-like protein 1-like	LGLFLLVFSFFFLLTWASFSF
Q12912	Lymphoid-restricted membrane protein (Protein Jaw1) [Cleaved into: Processed lymphoid-restricted membrane protein]	ALWLSIAFIVLFAALMSFLTG
P61566	Endogenous retrovirus group K member 24 Env polyprotein (Envelope polyprotein) (HERV-K101 envelope protein) (HERV-K_22q11.21 provirus ancestral Env polyprotein) [Cleaved into: Surface protein (SU); Transmembrane protein (TM)]	IGSTTIINLILILVCLFCLLL
P61567	Endogenous retrovirus group K member 7 Env polyprotein (Envelope polyprotein) (HERV-K(III) envelope protein) (HERV-K102 envelope protein) (HERV-K_1q22 provirus ancestral Env polyprotein) [Cleaved into: Surface protein (SU); Transmembrane protein (TM)]	IGSTTIINLILILVCLFCLLL
Q8N6L0	Protein KASH5 (Coiled-coil domain-containing protein 155) (KASH domain-containing protein 5)	LIPAPVLGLLLLLLLSVLLLG
Q8N6Q1	Transmembrane and coiled-coil domain-containing protein 5A	IFCCLFFITLFFIRLLSYMFF
Q12981	Vesicle transport protein SEC20 (BCL2/adenovirus E1B 19 kDa protein-interacting protein 1) (Transformation-related gene 8 protein) (TRG-8)	TDKLLIFLALALFLATVLYIV
P59773	UPF0258 protein KIAA1024-like	GLILLVVISILVTIVTIITFF
Q8WXH0	Nesprin-2 (KASH domain-containing protein 2) (KASH2) (Nuclear envelope spectrin repeat protein 2) (Nucleus and actin connecting element protein) (Protein NUANCE) (Synaptic nuclear envelope protein 2) (Syne-2)	AALPLQLLLLLLLLLACLLPS

Q8WVX3	Uncharacterized protein C4orf3 (Hepatitis C virus F protein-transactivated protein 1) (HCV F-transactivated protein 1)	WLDLWLFILFDVVVFLFVYFL
P32856	Syntaxin-2 (Epimorphin)	WIIIAVSVVLVAIIALIIGLSVGK
Q6IEE8	Schlafen family member 12-like	IFLFVCLFRFCLFVCWFVCFF
Q8NHP6	Motile sperm domain-containing protein 2	LLLSLTMLLLAFVTSFFYLLY
O95292	Vesicle-associated membrane protein-associated protein B/C (VAMP-B/VAMP-C) (VAMP-associated protein B/C) (VAP-B/VAP-C)	RLLALVVLFFIVGVIIGKIAL
Q96QK8	Small integral membrane protein 14	GISVTMILVAWMVIALILFLL
Q96JQ2	Calmin (Calponin-like transmembrane domain protein)	MMYFILFLWLLVYCLLLFPQL
Q71RC9	Small integral membrane protein 5	IVAFSVIILFTATVLLLLLIA
A2A2Y4	FERM domain-containing protein 3 (Band 4.1-like protein 4O) (Ovary type protein 4.1) (4.1O)	LLVVGLGLLLFVFPLLLLLLE
O42043	Endogenous retrovirus group K member 18 Env polyprotein (Envelope polyprotein) (HERV-K(C1a) envelope protein) (HERV-K110 envelope protein) (HERV-K18 envelope protein) (HERV-K18 superantigen) (HERV-K_1q23.3 provirus ancestral Env polyprotein) (IDDMK1,2 22 envelope protein) (IDDMK1,2 22 superantigen) [Cleaved into: Surface protein (SU); Transmembrane protein (TM)]	IRSTMIINLILIVVCLFCLLL
P50402	Emerin	VPLWGQLLLFLVFVIVLFFIY
Q96D59	Probable E3 ubiquitin-protein ligase RNF183 (EC 2.3.2.27)	IFAYLMAVILSVTLLLIFSIF

Q9P2W9	Syntaxin-18 (Cell growth-inhibiting gene 9 protein)	AGFRVWILFFLVMCSFSLLFL
Q01629	Interferon-induced transmembrane protein 2 (Dispanin subfamily A member 2c) (DSPA2c) (Interferon-inducible protein 1-8D)	IWALILGIFMTILLIIIPVLV
Q86Y82	Syntaxin-12	KKMCILVLVLSVIILILGLII
Q01628	Interferon-induced transmembrane protein 3 (Dispanin subfamily A member 2b) (DSPA2b) (Interferon-inducible protein 1-8U)	IWALILGILMTILLIVIPVLI
P0C2S0	Cortexin-2	TGFAFVGILCIFLGLLIIRCF
O75396	Vesicle-trafficking protein SEC22b (ER-Golgi SNARE of 24 kDa) (ERS-24) (ERS24) (SEC22 vesicle-trafficking protein homolog B) (SEC22 vesicle-trafficking protein-like 1)	KLAAVAVFFIMLIVYVRFWWL
P42167	Lamina-associated polypeptide 2, isoforms beta/gamma (Thymopoietin, isoforms beta/gamma) (TP beta/gamma) (Thymopoietin-related peptide isoforms beta/gamma) (TPRP isoforms beta/gamma) [Cleaved into: Thymopoietin (TP) (Splenin); Thymopentin (TP5)]	IPVWIKILLFVVVAVFLFLVYQAM
P13164	Interferon-induced transmembrane protein 1 (Dispanin subfamily A member 2a) (DSPA2a) (Interferon-induced protein 17) (Interferon-inducible protein 9-27) (Leu-13 antigen) (CD antigen CD225)	IWALILGILMTIGFILLLVFG
Q0VDE8	Adipogenin	FSFLVFWFCLPVGLLLLLIIW
Q9BZL3	Small integral membrane protein 3 (NGF-induced differentiation clone 67 protein) (Small membrane protein NID67)	IWVIVLIILATIVIMTSLLLC

Q96AJ9	Vesicle transport through interaction with t-SNAREs homolog 1A (Vesicle transport v-SNARE protein Vti1-like 2) (Vti1-rp2)	ILLVILGIIVVITILMAITFS
O75379	Vesicle-associated membrane protein 4 (VAMP-4)	IKAIMALVAAILLLVIIILIV
O95159	Zinc finger protein-like 1 (Zinc finger protein MCG4)	LLLLLGLLGFLALLALMSRLG
Q86Y07	Serine/threonine-protein kinase VRK2 (EC 2.7.11.1) (Vaccinia-related kinase 2)	VYYYRIIIPVLLMLVFLALFF
Q86W74	Ankyrin repeat domain-containing protein 46 (Ankyrin repeat small protein) (ANK-S)	LGFWRVLLLIFVIALLSLGIA
Q629K1	Triple QxxK/R motif-containing protein (Triple repetitive-sequence of QXXK/R protein homolog)	VGLVLAAILALLLAFYAFFYL
P0DKX4	Small integral membrane protein 18	CFVILLLFIFTVVSLVVLAFL
Q8N8F7	Leucine-rich single-pass membrane protein 1	VGLLIVLIVSLALVFFVIFLI
O15155	BET1 homolog (hBET1) (Golgi vesicular membrane-trafficking protein p18)	KLLCYMMLFSLFVFFIIYWII
P63027	Vesicle-associated membrane protein 2 (VAMP-2) (Synaptobrevin-2)	MMIILGVICAIILIIIIVYF
O15400	Syntaxin-7	CIIILILVIGVAIISLIIWGL
A2A2V5	Serine-rich and transmembrane domain-containing protein 1	IYVSIFLSLLAFLLLLLIIAL
Q6ZMZ3	Nesprin-3 (KASH domain-containing protein 3) (KASH3) (Nuclear envelope spectrin repeat protein 3)	VALPLQLLLLLFLLLLFLLPI
O60499	Syntaxin-10 (Syn10)	WCAIAVLVGVLLLVLILLFSL
P60606	Cortexin-1	TVFAFVLCLLVVLVLLMVRCV
Q13277	Syntaxin-3	LIIIIVLVVVLLGILALIIGL

O95183	Vesicle-associated membrane protein 5 (VAMP-5) (Myobrevin)	VGLVVVGVLLIILIVLLVVFL
Q7Z6J6	FERM domain-containing protein 5	LLLVTMGLLFVLLLLLIILTE
Q15836	Vesicle-associated membrane protein 3 (VAMP-3) (Cellubrevin) (CEB) (Synaptobrevin-3)	MWAIGITVLVIFIIIIIVWVV
Q4LDR2	Cortexin-3 (Kidney and brain-expressed protein)	MTFVFVILLFIFLGILIVRCF
A9Z1Z3	Fer-1-like protein 4	LVLLLLVLLTVFLLLVFYTIP
Q9HCU5	Prolactin regulatory element-binding protein (Mammalian guanine nucleotide exchange factor mSec12)	VPVWLLLLLCVGLIIVTILLL
Q8N112	Leucine-rich single-pass membrane protein 2	GFLLLLALLVLTCLVLALLAV
Q13190	Syntaxin-5	WLMVKIFLILIVFFIIFVVFL
P0DI80	Small integral membrane protein 6	LAVIILFITAVLLLILFAIVF
Q96F15	GTPase IMAP family member 5 (Immunity-associated nucleotide 4-like 1 protein) (Immunity-associated nucleotide 5 protein) (IAN-5) (hIAN5) (Immunity-associated protein 3)	IFVFLLLCSILFFIIFLFIFH
Q9NX77	Endogenous retrovirus group K member 13-1 Env polyprotein (Envelope polyprotein) (HERV-K_16p13.3 provirus ancestral Env polyprotein) [Cleaved into: Surface protein (SU); Transmembrane protein (TM)]	GSLLLLALLILVCLCCLLLVC
Q8N205	Nesprin-4 (KASH domain-containing protein 4) (KASH4) (Nuclear envelope spectrin repeat protein 4)	FLLILFLLFLLLVGAMFLLPA
P26678	Cardiac phospholamban (PLB)	FINFCLILICLLLICIIVMLL
O14662	Syntaxin-16 (Syn16)	MLVILILFVIIIVLIVVLVGV

Q9BV40	Vesicle-associated membrane protein 8 (VAMP-8) (Endobrevin) (EDB)	MIVLICVIVFIIILFIVLFAT
Q9NZ43	Vesicle transport protein USE1 (Putative MAPK-activating protein PM26) (USE1-like protein) (p31)	WLLWAMLIIVCFIFISMILFI
O43752	Syntaxin-6	WCAIAILFAVLLVVLILFLVL
Q9NZM1	Myoferlin (Fer-1-like protein 3)	WVIIGLLFLLILLLFVAVLLY
O75923	Dysferlin (Dystrophy-associated fer-1-like protein) (Fer-1-like protein 1)	IILFIILFILLLFLAIFIYAF
Q2WGJ9	Fer-1-like protein 6	IIIAFILIILIIFLVLFIYTL
Q9HC10	Otoferlin (Fer-1-like protein 2)	WLLLKLLLLLLLLLLLALFLY

TMD: transmembrane domain.

Supplemental Table 4. Animal models for ASNA1-mediated TA protein insertion related genes.

Gene	Synonyms	Species	Genotype	Mechanism	Phenotype	Refs
WRB	*CHD5, GET1*	*Mus musculus*	$Wrb^{tm1.1(KOMP)Vlcg}/Wrb^{tm1.1(KOMP)Vlcg}$	homozygous ko	embryonic lethality (<E9.5)	15
		Mus musculus	$Wrb^{tm1.1(KOMP)Vlcg}$/Wrb+	heterozygous ko	abnormal brain development	15
		Mus musculus	$Wrb^{fl/fl}$:Vglut3-Cre<br>$Wrb^{fl/fl}$:Vglut3-ires-Cre	conditional ko	progressive hearing impairment, tonic-clonic seizures	16
		Danio rerio	$wrb^{hi1482Tg}$	homozygous ko	abnormal myocardial repolarization, bradycardia	17
		Danio rerio	$wrb^{hi1482Tg/hi1482Tg}$<br>$wrb^{hi1482Tg/hi1482Tg}$; nl1Tg	homozygous ko	reduced auditory startle response, reduced visual evoked potentials	18
		Danio rerio	lri48Tg ; $wrb^{hi1482Tg}$<br>q16aTg ; q16bTg ; $wrb^{hi1482Tg/hi1482Tg}$<br>$wrb^{hi1482Tg/hi1482Tg}$	homozygous ko	photoreceptor synapse defects	19
		Danio rerio	$wrb^{hi1482Tg/hi1482Tg}$	homozygous ko	impaired hair cell exocytosis and hearing	16
		Oryzias latipalis	WT + MO chd5 (ATG)	knockdown	cardiac looping defects, abnormal chamber differentiation, ocular abnormalities	20

		Xenopus tropicalis	Tg (actc1:GFP)^{Mohun} + MO chd5 (ATG)<br>Tg (actc1:GFP)^{Mohun} + MO chd5 (SB)	knockdown	cardiac looping defects, abnormal chamber differentiation	[21]
CAMLG	*CAML, GET2*	*Mus musculus*	Caml^{tm1Rjb}/Caml^{tm1Rjb}	homozygous ko	embryonic lethality (E4.5-E7.5)	[22]
		Mus musculus	Caml^{tm1Rjb}/Caml^{tm2Rjb}	conditional ko	abnormal T-cell development	[23]
ASNA1	*TRC40, GET3*	*Mus musculus*	Asna1^{tm1Hbha}/Asna1^{tm1Hbha}	homozygous ko	embryonic lethality (E3.5-E8.5)	[24]
		Danio rerio	q16aTg ; q16bTg + MO1 asna1<br>WT + MO1 asna1 (ATG)<br>WT + MO2 asna1 (SB)	knockdown	decreased visual perception, photoreceptor synapse defects, lack of swim bladder	[19]
		Danio rerio	asna1$^{\Delta 7/\Delta 7}$	homozygous ko	impaired swim bladder inflation, decreased blood flow in dorsal aorta, impaired cardiac contractility, early lethality (6-8 dpf)	This study

ATG, translation-blocking; dpf, days post fertilization; E, embryonic day; ko, knock-out; MO, morpholino; SB, splice-blocking; WT, wild-type.

SUPPLEMENTAL FIGURES

Supplemental Figure 1. CRISPR/Cas9-induced *asna1* deletion in zebrafish.

(**A**) Schematic representation of guide RNA target site (*asna1* exon 5). Protospacer is highlighted in cyan; PAM in red. Bsrl recognition site used for genotyping is underlined. (**B**) Sequence and position of induced 7 bp deletion (Δ7) predicted to result in a frameshift and premature stop codon. (**C**) Chromatogram of PCR-amplified DNA from F1 fish showing wild-type and mutant sequence (reverse complement). The arrow indicates the position of the deletion.

A

5′-GACATGAATGCAGATCAGCTGGCGTCCAAACTGGAGGAGACGCTGCCTGTCATCCGCTCCGTCAGCGAGCAG-3′

B

5′-GACATGAATGCAGATCAGCTGGCGTCCAAACTGGAGGAGACGCTGCCTGTCATCCGCTCCGTCAGCGAGCAG-3′ *WT*

D M N A D Q L A S K L E E T L P V I R S V S E Q

5′-GACATGAATGCAGATCAGCTGGCG-------TGGAGGAGACGCTGCCTGTCATCCGCTCCGTCAGCGAGCAG-3′ *Δ7*

D M N A D Q L A W R R R C L S S A P S (missing 48 AA)

C

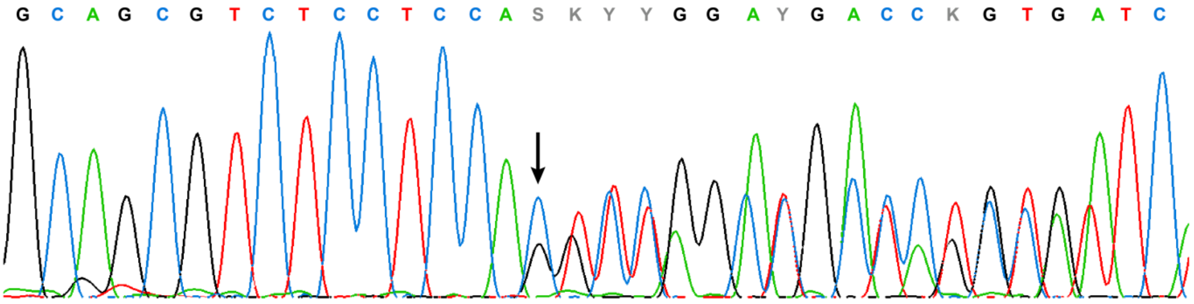

Supplemental Figure 2. Lateral views of the heart in wild-type and mutant zebrafish.

Microscopic images of the heart in wild-type and *asna1*$^{\Delta 7/\Delta 7}$ zebrafish larvae. The atria and ventricles are marked as A and V, respectively. The bulbus arteriosus (outflow tract) is marked as BA.

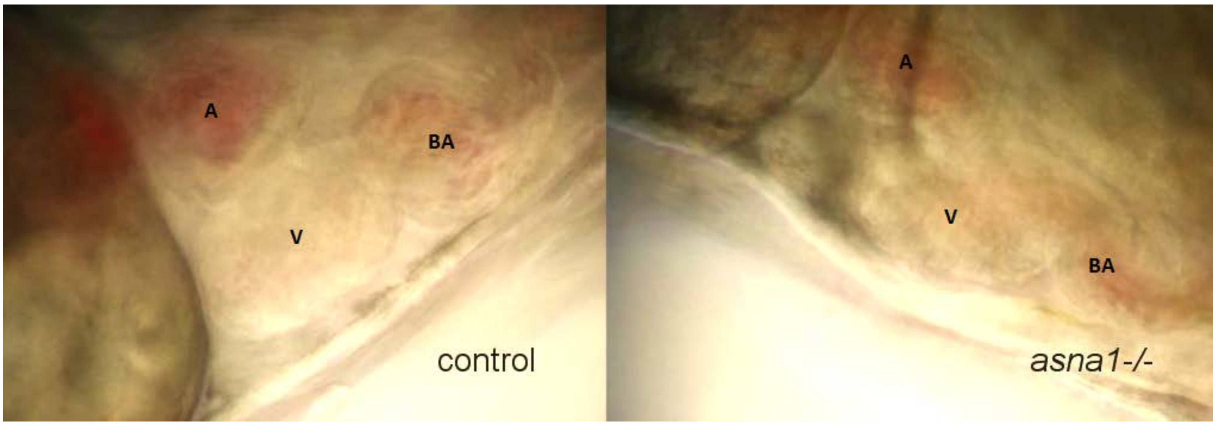

Supplemental Figure 3. M-mode imaging in both patients.

M-mode image of the heart in parasternal long axis view in (**A**) patient II:2 and (**B**) patient II:3 showing severely reduced left ventricular contractility.

A

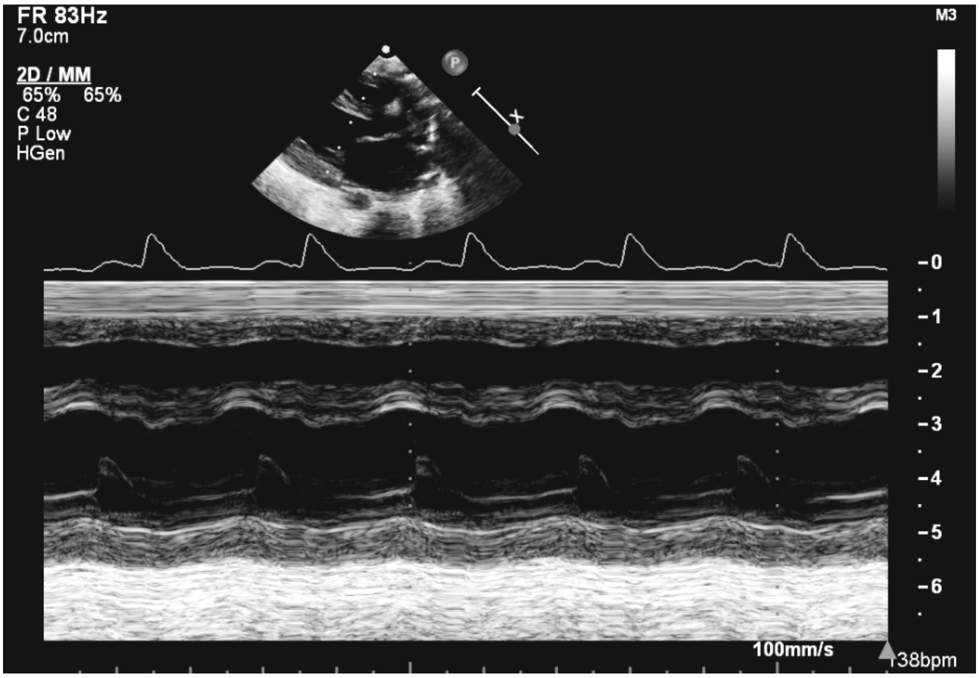

B

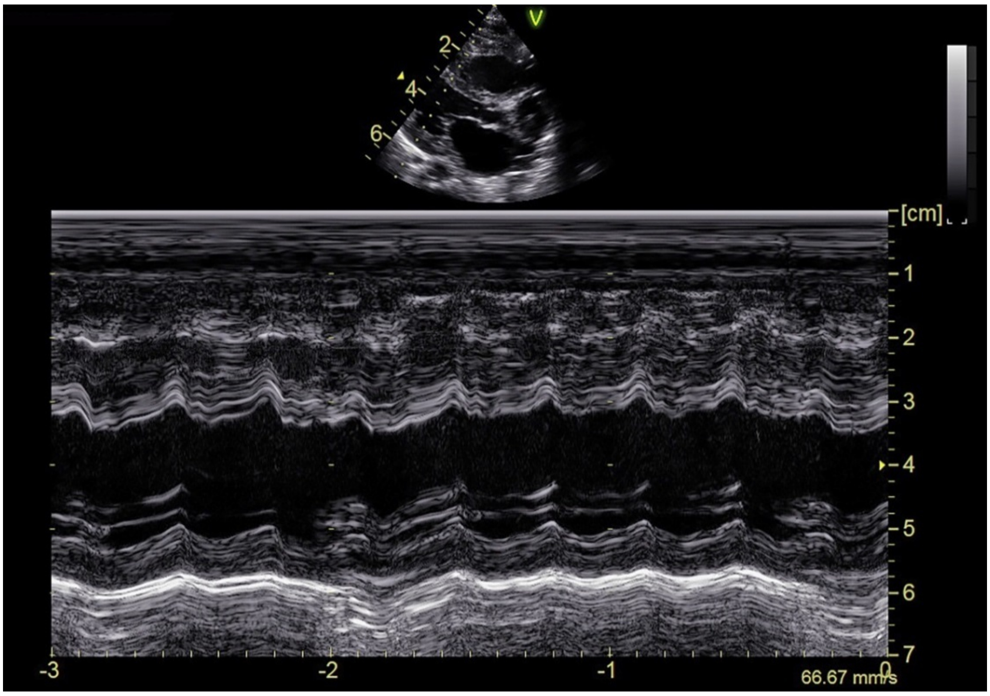

Supplemental Figure 4. Electrocardiography recordings of both patients.

(**A**) ECG of patient II:2 during hospital admission showing sinus rhythm at a rate of 130/min with extremely broad QRS complexes of 220 ms and normal QRS axis of 60 degrees. (**B**) ECG of patient II:3 during cardiopulmonary resuscitation (no prior ECG available).

A

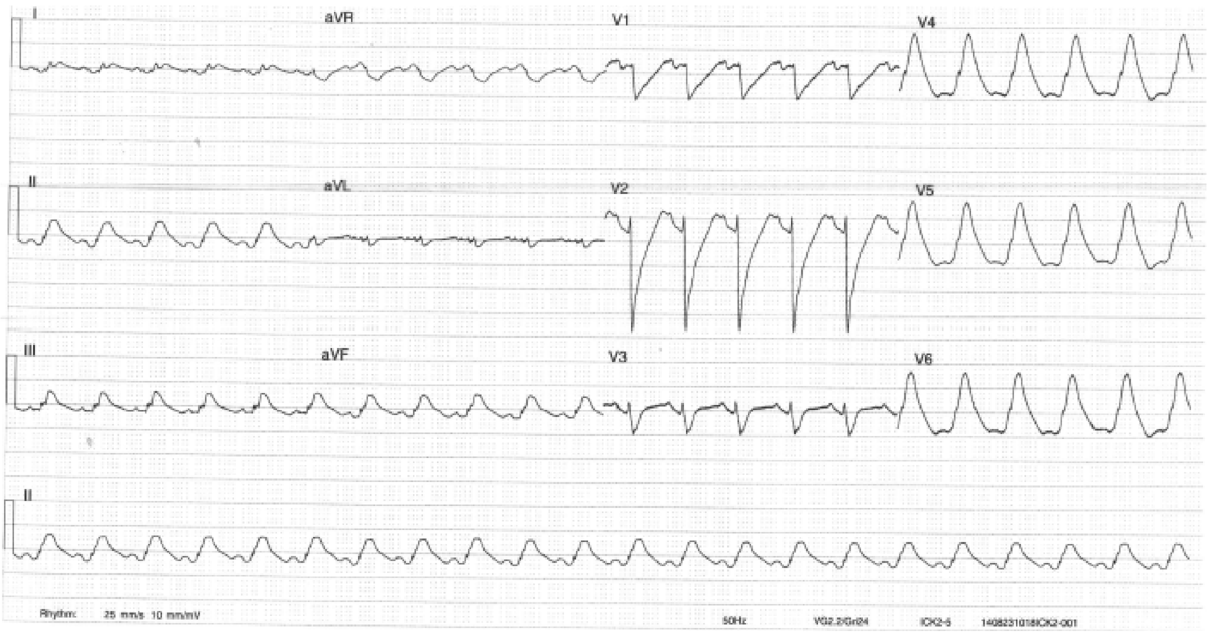

B

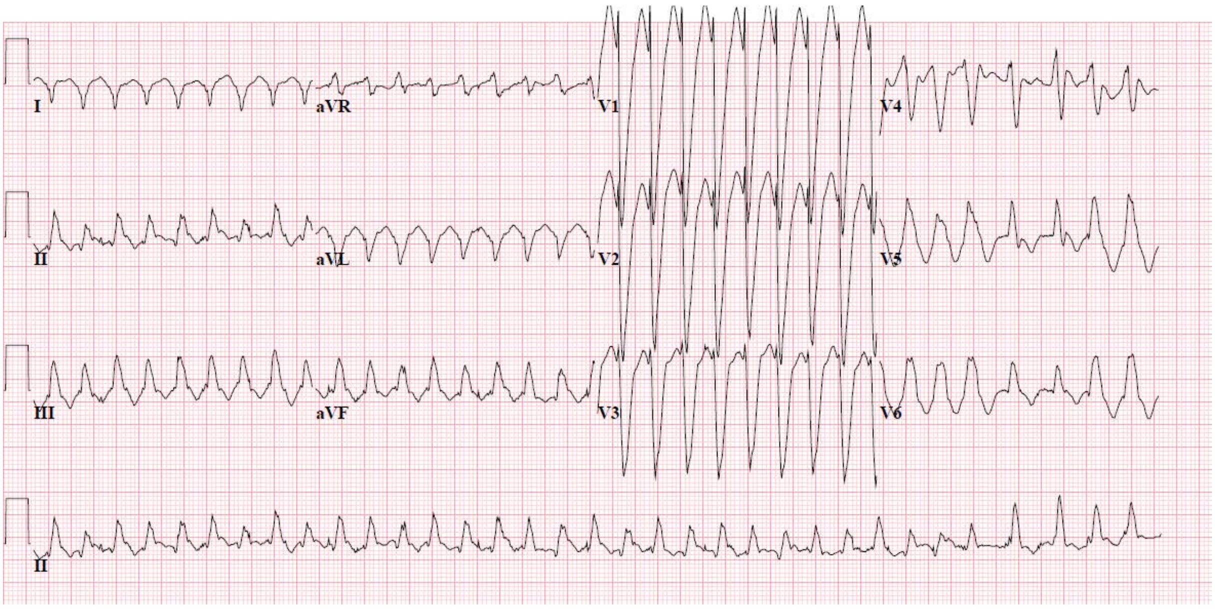

Supplemental Figure 5. Recombinant expression and purification of ASNA1 from *E. coli.*

(**A**) Expression tests of *E. coli* transformed with plasmids encoding either wild-type ASNA1 or the Val163Ala mutant. In each case, equal numbers of cells harvested before or after induction with 1 mM IPTG (for 3 hours at 37°C) were analyzed by SDS-PAGE and staining with Coomassie Blue. Two individual isolates of wild-type and four of mutant ASNA1 all show comparable expression levels of recombinant ASNA1 (indicated by the arrow). (**B**) The cells from a larger scale induction of wild-type and mutant ASNA1 (as in panel A) were collected, lysed by sonication, and subjected to chromatography using Ni-NTA columns. The total cells, soluble lysate, flow through, and elution fractions are shown. Note that a substantially higher proportion of wild-type ASNA1 is produced as a soluble protein, and recovered by chromatography. This is a consistent effect observed in more than six independent trials. (**C**) Increasing amounts of purified wild-type or mutant ASNA1 (ranging from 100 ng to 1 µg protein) were analyzed by SDS-PAGE and Coomassie staining to document concentration and purity. (**D**) A model TA protein containing the transmembrane domain from VAMP2 was translated in a purified *E. coli*-based translation system.[25] This system contains only recombinant translation factors and ribosomes, with no additional proteins. In addition, it contains ^{35}S-methionine to label the newly synthesized TA protein, and the photo-crosslinking amino acid benzyl-phenylalanine (BPA) and components for its incorporation at amber codons. A single amber codon in the transmembrane domain of the TA protein is used to incorporate this photo-crosslinking amino acid. The translation was supplemented with either wild-type or mutant ASNA1, which forms a complex with the newly made TA protein. The successful formation of the TA-ASNA1 complex was verified by UV irradiation to induce a covalent crosslink between these two proteins (indicated by "x ASNA1"). These recombinant TA-ASNA1 complexes were used for the insertion assay shown in Figure 4D.

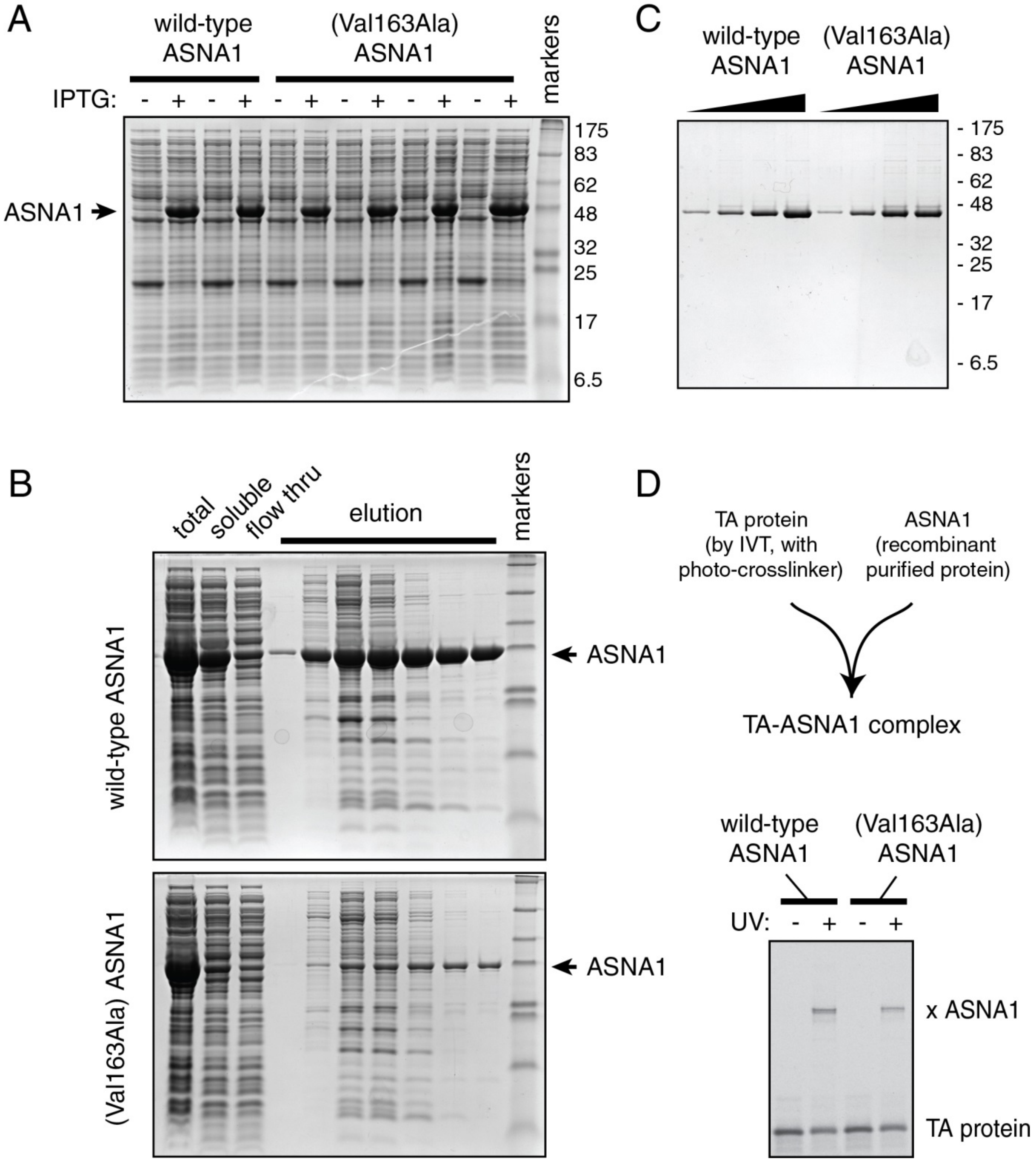
A
wild-type ASNA1
(Val163Ala) ASNA1
markers
IPTG: - + - + - + - + - + - +
ASNA1
175
83
62
48
32
25
17
6.5
C
wild-type ASNA1
(Val163Ala) ASNA1
- 175
- 83
- 62
- 48
- 32
- 25
- 17
- 6.5
B
total
soluble
flow thru
elution
markers
wild-type ASNA1
(Val163Ala) ASNA1
ASNA1
ASNA1
D
TA protein (by IVT, with photo-crosslinker)
ASNA1 (recombinant purified protein)
TA-ASNA1 complex
wild-type ASNA1
(Val163Ala) ASNA1
UV: - + - +
x ASNA1
TA protein

SUPPLEMENTAL VIDEOS

1. Cardiac ultrasound examination in patient II:2 showing poor contractility and thrombus formation in the left ventricle prior to death at age 7 weeks.

2. Cardiac ultrasound examination in patient II:3 showing minor abnormalities at age 9 days.

3. Repeat examination in patient II:3 on day 12 showing ventricular dysfunction and dilatation.

4. Microscopic imaging of blood flow velocity in wild-type and *asna1*$^{\Delta 7/\Delta 7}$ zebrafish larvae.

5. Microscopic imaging of heart contractions in wild-type and *asna1*$^{\Delta 7/\Delta 7}$ zebrafish larvae.

SUPPLEMENTAL REFERENCES

1. Pinto YM, Elliott PM, Arbustini E, Adler Y, Anastasakis A, Bohm M, et al. Proposal for a revised definition of dilated cardiomyopathy, hypokinetic non-dilated cardiomyopathy, and its implications for clinical practice: a position statement of the ESC working group on myocardial and pericardial diseases. *Eur Heart J.* 2016;37:1850-1858.

2. Ponikowski P, Voors AA, Anker SD, Bueno H, Cleland JG, Coats AJ, et al. 2016 ESC Guidelines for the diagnosis and treatment of acute and chronic heart failure: The Task Force for the diagnosis and treatment of acute and chronic heart failure of the European Society of Cardiology (ESC)Developed with the special contribution of the Heart Failure Association (HFA) of the ESC. *Eur Heart J.* 2016;37:2129-2200.

3. Li H, Durbin R. Fast and accurate long-read alignment with Burrows-Wheeler transform. *Bioinformatics.* 2010;26:589-595.

4. McKenna A, Hanna M, Banks E, Sivachenko A, Cibulskis K, Kernytsky A, et al. The Genome Analysis Toolkit: a MapReduce framework for analyzing next-generation DNA sequencing data. *Genome Res.* 2010;20:1297-1303.

5. Stefanovic S, Hegde RS. Identification of a targeting factor for posttranslational membrane protein insertion into the ER. *Cell.* 2007;128:1147-1159.

6. van Veen TA, van Rijen HV, Wiegerinck RF, Opthof T, Colbert MC, Clement S, et al. Remodeling of gap junctions in mouse hearts hypertrophied by forced retinoic acid signaling. *J Mol Cell Cardiol.* 2002;34:1411-1423.

7. Shao S, Rodrigo-Brenni MC, Kivlen MH, Hegde RS. Mechanistic basis for a molecular triage reaction. *Science.* 2017;355:298-302.

8. Mateja A, Szlachcic A, Downing ME, Dobosz M, Mariappan M, Hegde RS, et al. The structural basis of tail-anchored membrane protein recognition by Get3. *Nature.* 2009;461:361-366.

9. Guna A, Volkmar N, Christianson JC, Hegde RS. The ER membrane protein complex is a transmembrane domain insertase. *Science.* 2018;359:470-473.

10. Kuil LE, Oosterhof N, Geurts SN, van der Linde HC, Meijering E, van Ham TJ. Reverse genetic screen reveals that Il34 facilitates yolk sac macrophage distribution and seeding of the brain. *bioRxiv.* 2018:406553.

11. D'Astolfo DS, Pagliero RJ, Pras A, Karthaus WR, Clevers H, Prasad V, et al. Efficient intracellular delivery of native proteins. *Cell.* 2015;161:674-690.

12. Brinkman EK, Chen T, Amendola M, van Steensel B. Easy quantitative assessment of genome editing by sequence trace decomposition. *Nucleic Acids Res.* 2014;42:e168.

13. Hoage T, Ding Y, Xu X. Quantifying cardiac functions in embryonic and adult zebrafish. *Methods Mol Biol.* 2012;843:11-20.

14. The UniProt Consortium. UniProt: the universal protein knowledgebase. *Nucleic Acids Res.* 2017;45:D158-D169.

15. Blake JA, Eppig JT, Kadin JA, Richardson JE, Smith CL, Bult CJ, et al. Mouse Genome Database (MGD)-2017: community knowledge resource for the laboratory mouse. *Nucleic Acids Res.* 2017;45:D723-D729.

16. Vogl C, Panou I, Yamanbaeva G, Wichmann C, Mangosing SJ, Vilardi F, et al. Tryptophan-rich basic protein (WRB) mediates insertion of the tail-anchored protein otoferlin and is required for hair cell exocytosis and hearing. *Embo J.* 2016;35:2536-2552.

17. Milan DJ, Kim AM, Winterfield JR, Jones IL, Pfeufer A, Sanna S, et al. Drug-sensitized zebrafish screen identifies multiple genes, including GINS3, as regulators of myocardial repolarization. *Circulation.* 2009;120:553-559.

18. Lin SY, Vollrath MA, Mangosing S, Shen J, Cardenas E, Corey DP. The zebrafish pinball wizard gene encodes WRB, a tail-anchored-protein receptor essential for inner-ear hair cells and retinal photoreceptors. *J Physiol.* 2016;594:895-914.

19. Daniele LL, Emran F, Lobo GP, Gaivin RJ, Perkins BD. Mutation of wrb, a Component of the Guided Entry of Tail-Anchored Protein Pathway, Disrupts Photoreceptor Synapse Structure and Function. *Invest Ophthalmol Vis Sci.* 2016;57:2942-2954.

20. Murata K, Degmetich S, Kinoshita M, Shimada E. Expression of the congenital heart disease 5/tryptophan rich basic protein homologue gene during heart development in medaka fish, Oryzias latipes. *Dev Growth Differ.* 2009;51:95-107.

21. Sojka S, Amin NM, Gibbs D, Christine KS, Charpentier MS, Conlon FL. Congenital heart disease protein 5 associates with CASZ1 to maintain myocardial tissue integrity. *Development.* 2014;141:3040-3049.

22. Tran DD, Russell HR, Sutor SL, van Deursen J, Bram RJ. CAML is required for efficient EGF receptor recycling. *Dev Cell.* 2003;5:245-256.

23. Tran DD, Edgar CE, Heckman KL, Sutor SL, Huntoon CJ, van Deursen J, et al. CAML is a p56Lck-interacting protein that is required for thymocyte development. *Immunity.* 2005;23:139-152.

24. Mukhopadhyay R, Ho YS, Swiatek PJ, Rosen BP, Bhattacharjee H. Targeted disruption of the mouse Asna1 gene results in embryonic lethality. *FEBS Lett.* 2006;580:3889-3894.

25. Shimizu Y, Ueda T. PURE technology. *Methods Mol Biol.* 2010;607:11-21.
